# Domain swapping and *SMYD1* interactions with the PWWP domain of human hepatoma-derived growth factor

**DOI:** 10.1038/s41598-017-18510-8

**Published:** 2018-01-10

**Authors:** Li-Ying Chen, Yen-Chieh Huang, Shih-Tsung Huang, Yin-Cheng Hsieh, Hong-Hsiang Guan, Nai-Chi Chen, Phimonphan Chuankhayan, Masato Yoshimura, Ming-Hong Tai, Chun-Jung Chen

**Affiliations:** 1 Institute of Biotechnology, National Cheng Kung University, Tainan, 701 Taiwan; 2 Department of Biotechnology and Bioindustry Sciences, National Cheng Kung University, Tainan, 701 Taiwan; 3 Life Science Group, Scientific Research Division, National Synchrotron Radiation Research Center, Hsinchu, 30076 Taiwan; 4 Doctoral Degree Program in Marine Biotechnology, National Sun Yat-Sen University, Kaohsiung, 804 Taiwan; 5 Doctoral Degree Program in Marine Biotechnology, Academia Sinica, Taipei, 11529 Taiwan; 6 Institute of Biomedical Sciences, National Sun Yat-Sen University, Kaohsiung, 804 Taiwan; 7 Department of Physics, National Tsing Hua University, Hsinchu, 30013 Taiwan

## Abstract

The human hepatoma-derived growth factor (HDGF), containing the chromatin-associated N-terminal PWWP domain capable of binding the *SMYD1* promoter, participates in various cellular processes and is involved in human cancers. We report the first crystal structures of the human HDGF PWWP domain (residues 1–100) in a complex with *SMYD1* of 10 bp at 2.84 Å resolution and its *apo* form at 3.3 Å, respectively. The structure of the *apo* PWWP domain comprises mainly four β-strands and two α-helices. The PWWP domain undergoes domain swapping to dramatically transform its secondary structures, altering the overall conformation from monomeric globular folding into an extended dimeric structure upon DNA binding. The flexible loop2, as a hinge loop with the partially built structure in the *apo* PWWP domain, notably refolds into a visible and stable α-helix in the DNA complex. The swapped PWWP domain interacts with the minor grooves of the DNA through residues Lys19, Gly22, Arg79 and Lys80 in varied ways on loops 1 and 4 of the two chains, and the structure becomes more rigid than the *apo* form. These novel structural findings, together with physiological and activity assays of HDGF and the PWWP domain, provide new insights into the DNA-binding mechanism of HDGF during nucleosomal functions.

## Introduction

Human hepatoma-derived growth factor (HDGF) is highly expressed in the developing heart, tumour cell lines and normal tissue ubiquitously with mitogenic and angiogenic activities^1–3^. Previous studies have demonstrated that HDGF participates in various cellular processes, such as the formation of vascular lesion^4^, astrocyte proliferation^5^ and cardiovascular differentiation^1^. Over-expressed HDGF can be found in several type of human cancers, including hepatocellular carcinoma^6^, pancreatic cancer^7^, oral cancer^8^, breast cancer^9^ and non-small cell lung cancer^9^. Human HDGF has also been shown to be involved in the phosphatidylinositol 3-kinase (PI3K)/Akt pathway, which is related to breast cancer and hepatocellular carcinoma. This pathway regulates post-translational histone modification^6,10,11^. Moreover, HDGF is involved in cardiovascular development and transcriptional regulation, resulting in its function as a transcriptional repressor^3,12^.

HDGF generally comprises two portions, an N-terminal domain with a highly conserved PWWP motif (residues 1–100 for human HDGF) and a variable C-terminal domain (residues 101–240)^13^. These two domains play distinct roles in physiology. The dominant nuclear location signal 2 (NLS2) located in the C-terminus in HDGF might assist the translocation of HDGF into the nucleus and subsequently regulates its mitogenic activity^14,15^. The PWWP motif of the N-terminal domain (PWWP domain) is named after the Pro-Trp-Trp-Pro motif, but only the third (Trp) and fourth (Pro) residues are highly conserved among various PWWP domain-containing proteins, whereas the first and second residues are variable^16^. The sequence of the PWWP motif of human HDGF is _24_Pro-His-Trp-Pro_27_, of which the second residue is variable. The highly conserved PWWP domains are discovered in numerous eukaryotes, from unicellular organisms to humans. For instance, bromo and plant homeodomain finger–containing protein 1 (brpf1) that promotes histone acetylation utilizes its C-terminal PWWP domain to recognize histone modifications^17,18^. The mammalian DNA methyltransferase, DNMT3B, which contains the PWWP domain, is not only linked to human immunodeficiency, centromere instability and facial anomalies syndrome but also to distinct cancers^19–21^. Another PWWP-containing protein, Lens epithelium-derived growth factor (LEDGF/p75), plays an essential role in the integration of HIV-1 cDNA into human chromosomes^22–25^. To date, more than twenty PWWP domain-containing proteins from human genomes have been discovered, most of which are chromatin-associated^26^.

The PWWP domain can interact with heparin, a linear polymer consisting of repeating units of uronic acid and glucosamine residues on the cell surface^16,27^. The PWWP domain of HDGF may first bind to heparin through its positively charged residues and then be internalized into a cell^27,28^. In addition, nucleolin (NCL) located on the plasma membrane functions as a receptor for HDGF through interactions with of the HDGF PWWP domain^6^. Because of its chromatin-associated properties, the PWWP domain exhibits a methylated histone and DNA-binding capability^16,29,30^. The PWWP domain of DNA methyltransferase DNMT3B has been demonstrated to show no preference for double-stranded DNA (dsDNA), based on DNA binding assays^29,31^. Previous work has shown that the PWWP domain has greater binding affinities towards various types of dsDNA, instead of single-stranded DNA, indicating that the PWWP domain can interact with dsDNA nonspecifically^32^. In addition to the nonspecific DNA binding, the HDGF PWWP domain is capable of recognizing the *SMYD1* promoter (5′-CAGGCTGGTCTTGAACTCCTGACCTCAGATGATCCATG TGCCTCGGCCTCCCAAGGTGGGGATTACAGGCGTGAGCCACC-3′)^12^. HDGF has been demonstrated to bind to the *SMYD1* promoter containing a HDGF core-binding sequence with a shortest length of 37 bp from human chromatin and functions as a transcriptional repressor^3,12^. HDGF interacts with the *SMYD1* promoter through its N-terminal PWWP domain; however, the precise interaction of the PWWP domain with *SMYD1* remains unclear^12^.

Several structures of the PWWP domains of various proteins from different species have been reported using NMR and X-ray approaches, but they represent only either the *apo* form or complexes with histone-related peptides^16,28–30^. The NMR structures of a monomeric form of the *apo* soluble HDGF PWWP domain and one domain-swapped dimeric form of the *apo* refolded PWWP domain have been determined, respectively (PDB entries: 1RI0, 2NLU)^27,28^. Despite the preceding work, the mechanisms of DNA binding and interactions with the PWWP domain remain puzzling because of a lack of essential knowledge regarding the intact structure in complex with DNA molecules. Here, we report the first crystal structures of the human HDGF PWWP domain in complex with a 10-bp *SMYD1* and its unbound *apo* form. Our structural studies, with physiological assays, provide new insights into the PWWP-DNA interaction pattern, which might facilitate research into the role of the PWWP domain in nucleosomal functions.

## Results

### Overall structure of the HDGF *apo* PWWP domain

**Figure 1:**
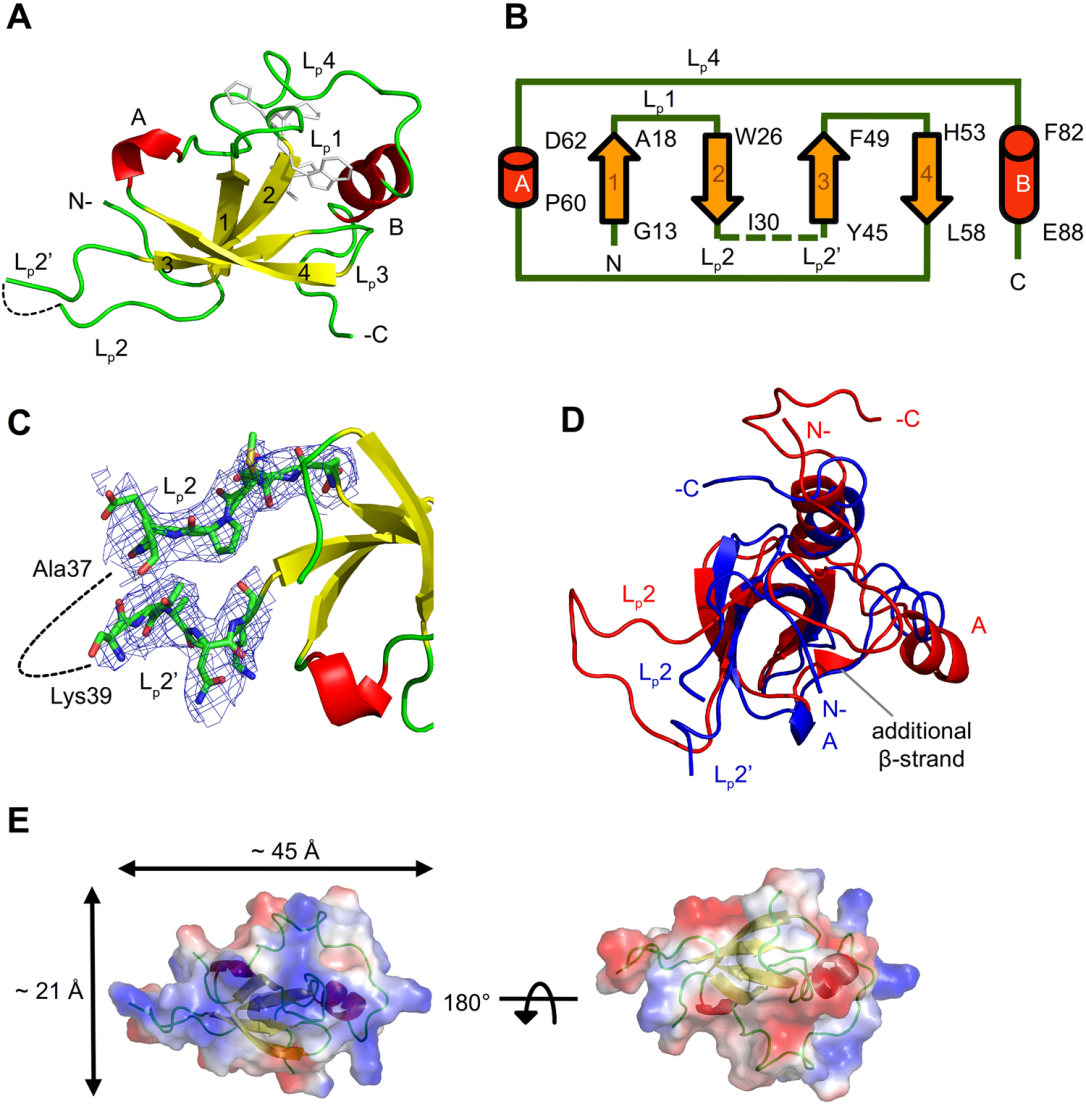
Crystal structure of the HDGF *apo* PWWP domain. (**A**) The monomer structure is shown as a ribbon. The disordered region (_37_AVK_39_) of loop2 is presented as a dashed line. The residues of the PWWP motif are shown in stick. (**B**) Topology of secondary structures of the *apo* PWWP domain. The sequence numbers are labeled. Arrow: β-strand; the cylinder: α-helix; solid line: loop; the dashed line: the disordered region of loop2. (**C**) The hinge loop, loop2, is devoid of electron density (blue mesh, 2*F*_o_-*F*_c_ at 0.8 σ) from Ala37 to Lys39 (dashed line). The residues Asp31 to Lys44 are shown as sticks. (**D**) Superposition of the crystal structure (blue) and the NMR solution structure (PDB entry: 1RI0, red) of the PWWP domain. The positions of the first defined αA helix are distinct. There is an additional β-strand in the NMR structure. The αA (Pro60 – Asp62) exists after β4 (His53 – Leu58) in the crystal structure, whereas αA (Tyr66 – Lys72) is defined after the additional short β-strand in the solution structure. (**E**) Charge distribution of the *apo* PWWP domain. One side of the molecular surface is equipped with positively charged region (blue), whereas the other side consists of slightly negatively charged (red) and neutral (white) region. The dimensions of the positively charged surface are ~45 × 21 Å^2^.

We obtained crystals of the *apo* PWWP domain after several crystallization attempts, and determined the crystal structure at 3.3 Å resolution (Table 1). Crystals belong to the hexagonal space group *P*6_4_22 with one PWWP domain in an asymmetric unit. The overall structure of the *apo* PWWP domain comprises mainly four β-strands and two α-helices. The truncated protein sequence consists of the first 100 amino acids of HDGF, but the first N-terminal eight residues are disordered without an interpretable electron density. The structure thus begins from a short N-terminal turn followed by two β-strands (β1 and β2) (Fig. 1A,B). The _24_PWWP_27_ motif is located on loop1 between the β1 and β2 strands. The structure of loop2, after the strand β2, is only partially built because of its diminished electron density from Ala37 to Lys39, indicating that this loop is flexible in the *apo* PWWP domain (Fig. 1C). The other two β-strands (β3 and β4) follow loop2, with one connecting loop linked to the C-terminal α-helices (αA and αΒ) and another connecting loop (Fig. 1A,B). The X-ray structure of the *apo* HDGF PWWP domain exhibits several notable structural features that differ from the NMR solution structure (PDB entry: 1RI0): the length and amounts of β-strands as well as the locations of the α-helices. One additional short β-strand (Leu63 – Phe64) exists in the solution structure. The positions of the defined first α-helix (αA) in the two structures are also distinct (Fig. 1D).

**Table 1:** Crystallographic data and refinement statistics of the HDGF *apo* PWWP domain and the PWWP-*SMYD1* complex.

Data collection	*apo* PWWP domain (PDB: 5XSL)	PWWP-SMYD1 complex (PDB: 5XSK)
Space group	*P*6_4_22	*P*3_1_
Cell dimensions		
*a*, *b*, *c* (Å)	79.5, 79.5, 105.1	32.4, 32.4, 205.8
Resolution (Å)	30–3.3 (3.42–3.3)^a^	30–2.84 (2.94–2.84)
*R*_merge_ (%)^b^	8.1 (43.8)	10.9 (58.5)
<I/σ>	21.89 (3.59)	17.25 (2.57)
CC_1/2_	0.99 (0.96)	0.96 (0.81)
Completeness (%)	100 (100)	98.7 (90.6)
Redundancy	12.8 (11.9)	8.3 (3)
**Refinement**		
Resolution (Å)	30–3.3 (3.42–3.3)	0–2.84 (2.94–2.84)
Unique reflections	3320	5853
*R*_work_/*R*_free_ (%)	22.5/28.2	19.1/26.9
No. atoms	702	1903
Protein	699	1477
Peptide/DNA	—	410
Ligand/ion	—	16
Water	3	—
B-factors (Å^2^)		
Protein	73.83	45.03
Peptide/DNA	—	58.79
Ligand/ion	—	40.28
Water	40.18	—
RMSD		
Bond length (Å)	0.01	0.01
Bond angles (°)	1.85	1.49

^a^Values in parentheses are for the highest resolution shell (3.42–3.3 Å in HDGF *apo* PWWP domain); (2.94–2.84 Å in HDGF PWWP-*SMYD1* complex).

^b^ *R*_merge_ = Σ_*hkl*_Σ_*j*_|*I*(*hkl;j*)−<*I*(*hkl*)>|/Σ_*hkl*_Σ_*j*_<*I*(*hkl;j*)>, where *I*(*hkl;j*)is the *j*th measurement of the intensity of the unique reflection (*hkl*), and *I*(*hkl*) is the mean overall symmetry related measurement.

RMSD: root mean square deviation.

An inspection of the distribution of surface charge of the *apo* PWWP domain reveals that the positively charged region and the slightly negatively charged and neutral region are separated on two opposite sides of the molecular surface (Fig. 1E). This charge distribution might result in the *apo* PWWP domain possessing a high value of p*I*, 9.19. The positively charged surface is generally thought to be the primary area interacting with the negatively charged DNA molecule, but our structure of the DNA complex shows an unexpected result and constitutes a detailed DNA-binding region, which is described in the following sections.

### Multiple forms of the HDGF *apo* PWWP domain

In general large-scale culture procedures, the *apo* PWWP domain could be collected from two fractions with distinct elution times in the size-exclusion chromatography, as confirmed by SDS-PAGE (Fig. S1A,S1B). These two fractions indicated that the *apo* PWWP domain could exhibit both monomeric and dimeric forms. The ratio of these two forms depended on the initial protein concentration during the size-exclusion chromatography. The *apo* PWWP domain is present as the monomer with an initial concentration of less than ~1.5 mg/mL, whereas the proportion of dimer increased as the concentration increases (Fig. S1C,S1D). Moreover, based on our observations, the dimeric PWWP domain in buffer containing the ionic-strength salt, such as NaCl (150 mM), could be transformed into the monomers within three days. In crystals of the *apo* PWWP domain, monomers related to crystallographic two-fold symmetries exhibit few interactions with residues on β4 and αB regions, respectively, with the average hydrogen-bond distance larger than 3.1 Å (Fig. S1E). Therefore, the weak interactions between monomers may occur when the concentration of the PWWP domain is increased and these contact monomers may be dissociated when the concentration decreases.

### Overall structure of the domain-swapped HDGF PWWP-*SMYD1* complex

**Figure 2:**
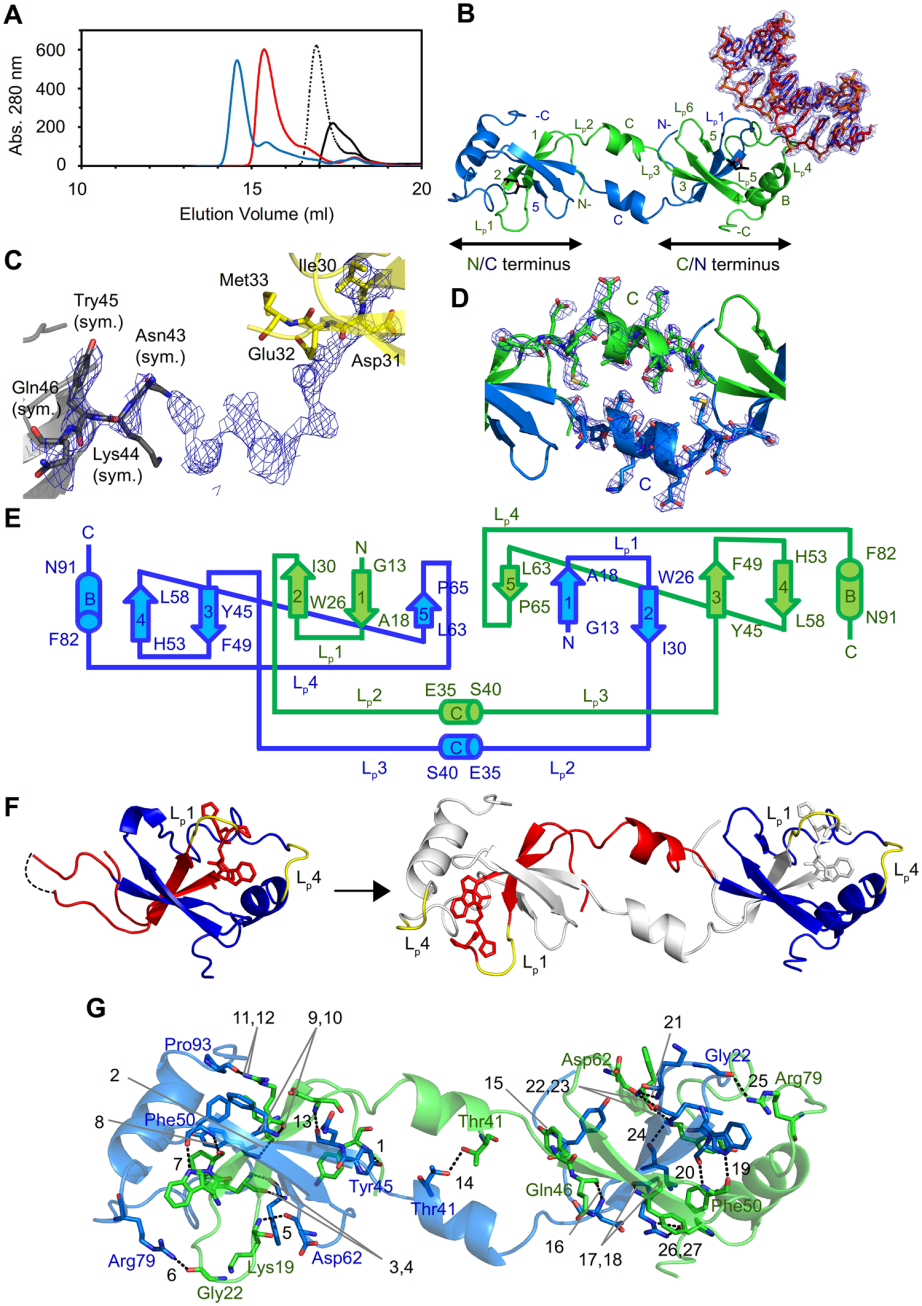
Overall structure of the HDGF PWWP-*SMYD1* complex. (**A**) Analyses of interactions of PWWP-*SMYD1* complexes with various DNA lengths by size-exclusion chromatography (Superdex-200). The elution volume was further decreased with a longer length of *SMYD1*. From small to large elution volume: the PWWP-15 bp *SMYD1* complex (blue line), PWWP-10 bp *SMYD1* (red line), PWWP-5 bp *SMYD1* (dashed line) and the *apo* PWWP domain (black line). (**B**) The domain-swapped structure of the PWWP-*SMYD1* complex comprises a protein dimer (ribbon) and a 10-bp *SMYD1* (orange stick) with interpretable density (blue mesh, 2*F*_o_-*F*_c_ at 1 σ). The swapped domain “N/C terminus” consists of the N-terminus of chain A (green) and the C-terminus of chain B (marine), whereas the other swapped domain “C/N terminus” is composed of the C-terminus of chain A and the N-terminus of chain B. Loop1 (chain B) and loop4 (chain A) in the C/N terminus are involved in *SMYD1* interactions. Two MPD molecules from the crystallization buffer are shown as black sticks (**C**) The unbiased initial density map (blue mesh, 2*F*_o_-*F*_c_ at 1.3 σ) with the globular structure template from the *apo* PWWP domain shows the continuous structural feature traceable from Asp31 of chain B to the symmetry-related Asn43 of chain A. (**D**) The unambiguous electron density of the omit map (blue mesh, 2*F*_o_-*F*_c_ at 1.3 σ) shows that the flexible loop2 in the globular monomeric *apo* PWWP domain is folded into the rigid αC helix (Gln35 – Ser40) to extend in a dimeric complex form. (**E**) The topology of secondary structures of the swapped PWWP-*SMYD1* complex. Arrow: β-strand, cylinder: α-helix, solid line: loop. (**F**) Schematic representation of the dimeric swapped PWWP structure. The involved swapped region is shown in red and the other regions are in blue in one monomer (left). The other molecule of a dimer is shown in grey (right). The residues of the PWWP motif are indicated in stick, whereas the DNA-binding loops are shown in yellow. (**G**) Dimeric interactions in the dimeric PWWP-*SMYD1* complex. The residues involved in interactions of domain swapping are shown as sticks with dashed lines between each other (chain A, green; chain B, marine). Numbers indicates the pair interactions are shown in Table S1.

Examination of the crystal structure of the *apo* PWWP domain reveals that the positively charged area on the surface of the *apo* PWWP domain covers ~45 Å in length that might accommodate and interact with DNA of a size up to 13 bp (Fig. 1E). We have therefore designed *SMYD1* of various lengths accordingly for complex formation. To facilitate crystallization of the HDGF PWWP-*SMYD1* complex, we have tested three *SMYD1* with sizes of 5, 10 and 15 bp. The complex formation is confirmed by size-exclusion chromatography; the elution profiles of these complexes with *SMYD1* of various lengths are all shifted compared to that of the *apo* PWWP domain (Fig. 2A). The respective fractions of various complexes are subsequently collected for crystallization. Among the three complexes, crystals of the PWWP complex with 10-bp *SMYD1* have been obtained. The crystal structure of the HDGF PWWP-*SMYD1* complex is determined at 2.84 Å resolution (Table 1). The asymmetric unit contains one swapped PWWP dimer and one 10-bp *SMYD1*, indicating that the PWWP domain dimerises with domain swapping to interact with one DNA molecule in the PWWP-*SMYD1* complex.

Based on the unambiguous electron density, the conformation of the PWWP domain in the complex has altered unexpectedly from a globular shape in the *apo* form structure to an extended shape (Figs 1A and 2B,C,D). The composition of the secondary structure contents in the PWWP-*SMYD1* complex is distinct from that in the *apo* PWWP domain (Figs 1B and 2E). In particular, the flexible loop2 with the partially built structure in the *apo* PWWP domain notably refolds into a visible α-helix (αC) based on the omit map (Figs 1C and 2C,D). This intrinsic flexible loop is apparently capable of exhibiting distinct conformations in both the monomer of the *apo* PWWP domain as well as the complex dimer, where it functions as a *hinge loop* ^33^. The C-terminus of one protein molecule (chain A) is elongated further after this newly formed αC helix and interacts tightly with the N-terminal region of the other molecule (chain B) to form a dimer, which undergoes *3D domain swapping* ^33^ (Fig. 2F). To explain the swapped domains in the following sections, we define one swapped domain consisting of the C-terminus from chain A and the N-terminus from chain B as the “swapped C/N terminus” and the other swapped domain as the “swapped N/C terminus” (Fig. 2B).

Several residues are involved in the dimer formation of the PWWP domain in the *SMYD1* complex. Thr41 located on each hinge loop from the A and B chains can interact with each other. Tyr10, Leu15, Phe17, Lys19, Gly22, Trp26, Pro27, Arg29 and Asp31 in chain A interact with Tyr45, Gln46, Phe48, Phe50, Asp62, Phe64, Tyr66, Arg79 and Pro93 in chain B to maintain the dimeric structure of the complex (Fig. 2G). Among these interacting residues, Arg29 presumably plays an important role in protein dimerization because it is involved in four hydrogen bonds through interactions with two residues, Phe48 and Pro93, whereas the other residues form only one or two hydrogen bonds. All residues involved in protein dimerization of the PWWP-*SMYD1* complex with the corresponding interaction distances are shown in Table S2.

### Interactions between the HDGF PWWP domain and 10-bp *SMYD1*

**Figure 3:**
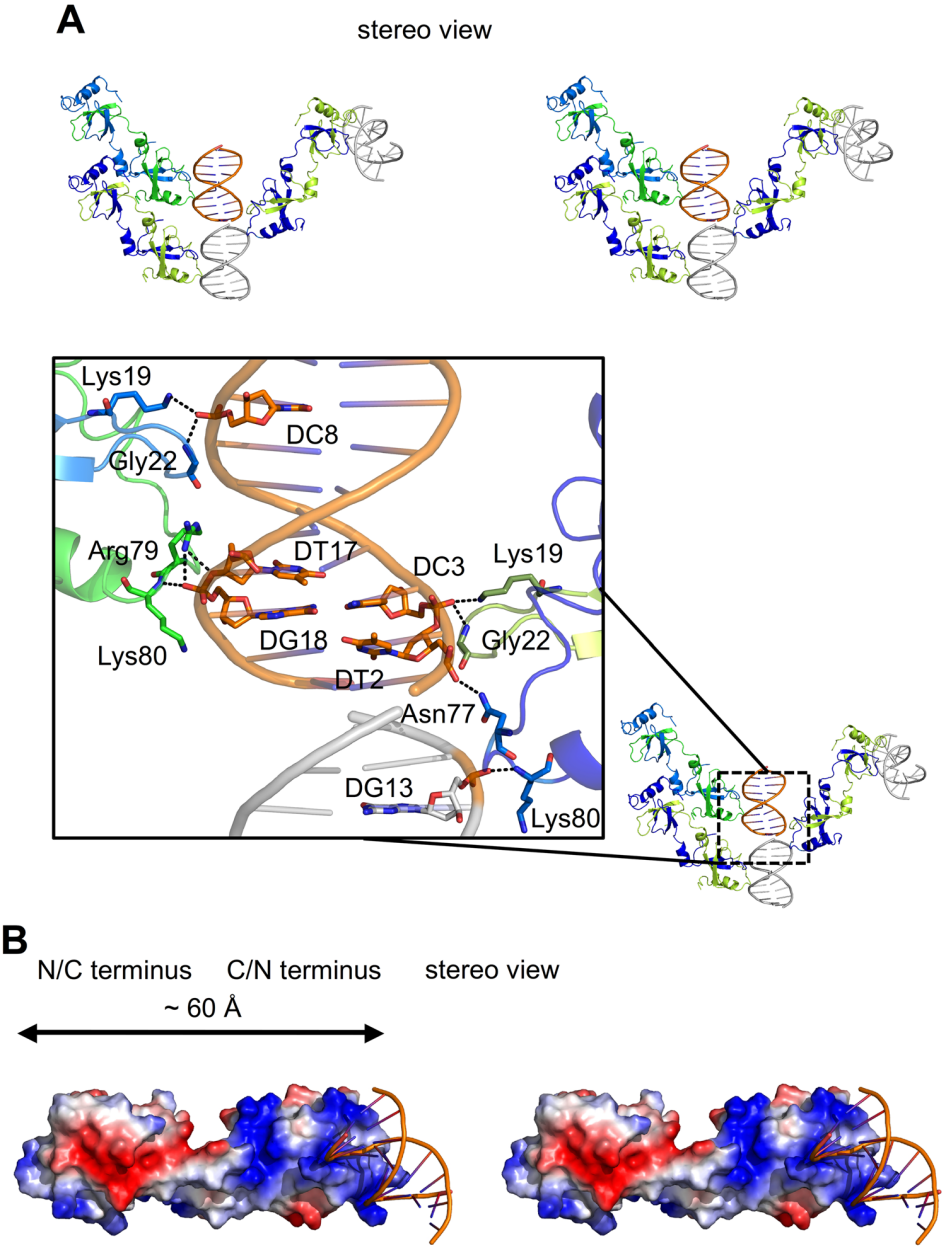
Interactions between the PWWP domain and *SMYD1*. (**A**) Stereo view of DNA binding to symmetry-related molecules (upper panel). The PWWP-*SMYD1* complex comprises chain A (green) and chain B (marine) and *SMYD1* (orange). The symmetric related chain A (limon), chain B (blue) and DNA (grey) are generated. The detailed interactions between DNA and residues are shown in the enlarged panel. Gly22 and Lys80 from both C/N and N/C termini interact with DNA with the main-chain N atoms; other residues contact to DNA through their side chains. Arg79 binds to DNA with two hydrogen bonds, whereas the others utilize one. The involved residues and base pairs are shown as sticks with interactions in dashed lines. (**B**) Stereo view of the surface-charge distribution of the PWWP-*SMYD1* complex. The positively charged region covers dimension of ~60 Å as measured from Arg79 to Gly22 with the main-chain structures, of which only the partial area at the end is utilized for DNA interactions.

Based on the structure of the HDGF PWWP-*SMYD1* complex, two major DNA-binding regions are identified: loop1 (_19_KMKG_22_) from chain B and loop4 (_77_NKRK_80_) from chain A in the swapped C/N terminus (Fig. 2B). Accordingly, chain A utilizes Arg79 and Lys80 on loop4 to interact with DNA; chain B uses Lys19 and Gly22 on loop1 to bind DNA at the C/N terminus (Fig. 3A).

Because of the pseudo two-fold symmetry within the structure of the HDGF PWWP-*SMYD1* complex, another potential DNA-binding region may exist on loop1 and loop4 of the swapped N/C terminus (Fig. 3A). Therefore, we have generated the symmetry-related PWWP-*SMYD1* complex molecules to illuminate the DNA-binding regions on swapped C/N and N/C termini in more detail. At the swapped N/C terminus, chain A interacts with DNA through Lys19 and Gly22, and chain B interacts with DNA via Asn77 and Lys80 (Fig. 3A). All residues involved in DNA interactions and their corresponding interaction distances are depicted in Table 2.

**Table 2:** Detailed interactions of each DNA-binding residue and the DNA nucleotide. The residues and DNA generated by crystallographic symmetry are labeled with “Sym.”.

Chain	PWWP domain		*SYMD1* DNA		Distance (Å)
	Residue	Atom	Nucleotide	Atom	
Chain A (loop4)	Arg 79	NH1	DT17	O3′	2.9
	Arg 79	NH1	DG18	OP1	3.0
	Lys 80	N	DG18	OP1	2.8
Chain B (loop1)	Lys 19	NZ	DC8	OP1	3.1
	Gly 22	N	DC8	OP1	3.0
Sym. Chain A (loop1)	Lys 19	NZ	DC3	OP1	2.9
	Gly 22	N	DC3	OP1	2.9
Sym. Chain B (loop4)	Asn 77	ND2	DT2	OP1	3.2
	Lys 80	N	DG13 (Sym.)	OP1	2.6

Considering both the swapped C/N and N/C termini, a total of eight residues participate in DNA interactions, including Lys19, Gly22, Arg79, Lys80 in chain A and Lys19, Gly22, Asn77, Lys80 in chain B. Among them, Gly22 and Lys80 in both chains contact DNA through the main-chain N atoms rather than their side chains. The PWWP domain thus may interact with DNA through residues with varied interactions in addition to the charge-charge interactions (Fig. 3A and Table 2). The distance (12.3 or 12.8 Å) tip-tip between loop1 and loop4 at the swapped C/N or N/C terminus is similar to the spatial dimension (~12 Å) of a minor groove of DNA (Fig. S2).

In summary, the HDGF PWWP domain utilizes loop1, which occurs before the PWWP motif, and loop4, before the αB helix, on both swapped C/N and N/C termini to interact with DNA. The molecular dimension of the swapped dimer with the positively charged surface of the PWWP-*SMYD1* complex is ~ 60 Å, but only the partial region is involved in DNA interactions (Fig. 3B). Together, the charge-charge interactions may thus play an essential role in facilitating the PWWP domain to approach DNA, and subsequently the residues with varied interactions of the swapped C/N and N/C termini interact and bind to DNA to form the stable PWWP-*SMYD1* complex (Fig. 3).

### Structural and *B*-factor comparisons of the PWWP-*SMYD1* complex and *apo* form

**Figure 4:**
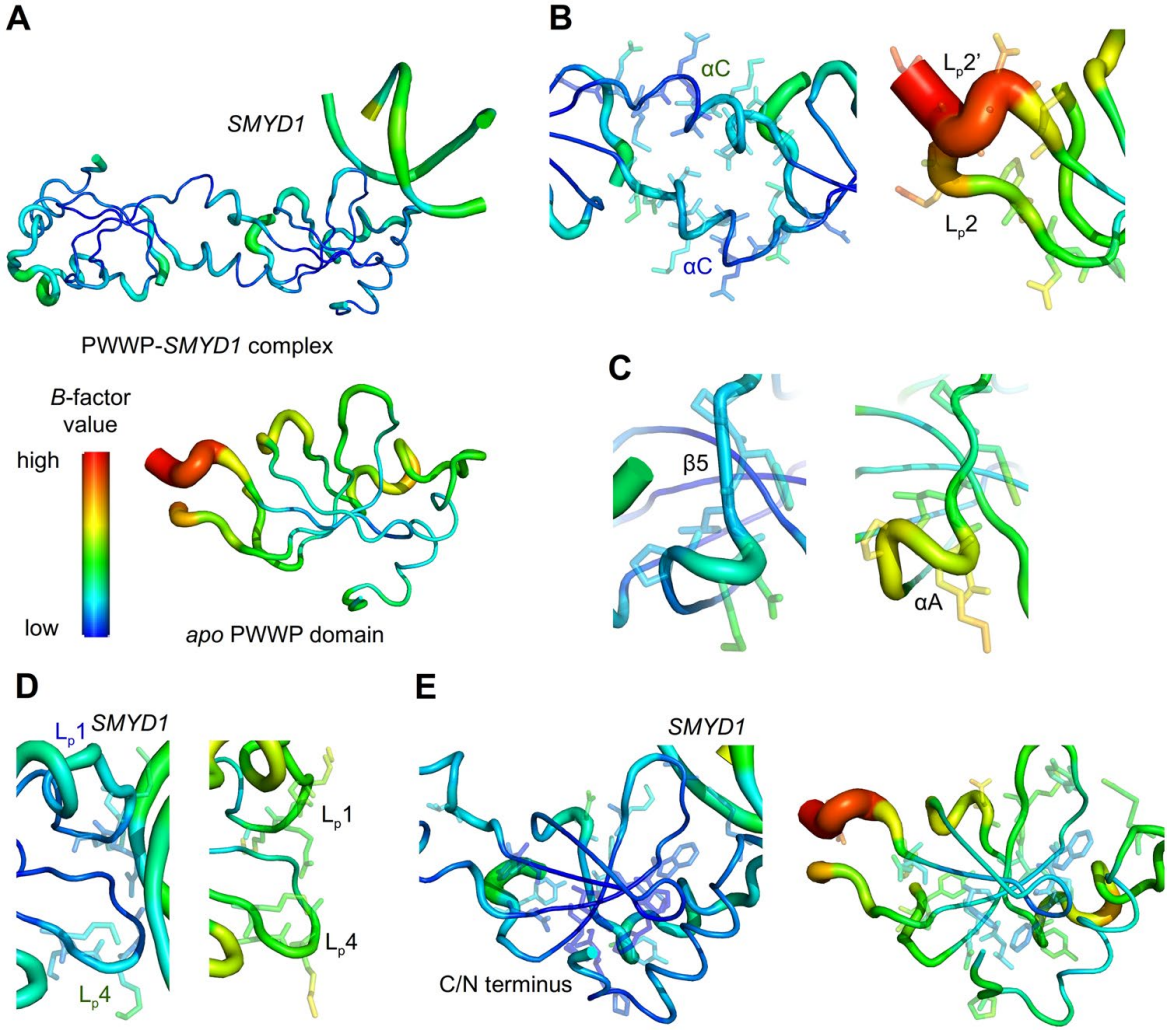
Comparisons of *B*-factors at regions with large structural differences between the complex and the *apo* forms. (**A**) The overall *B*-factor of the PWWP-*SMYD1* complex is less than that of the *apo* PWWP domain. (**B**) The *B*-factors of the residues on the hinge loop in the *apo* form (right panel) are much greater than that of the newly formed αC in the complex (left). (**C**) The *B*-factors of the region with the deformed αA (Pro60 – Asp62) and newly formed β5 (Leu63 – Pro65) in the PWWP-*SMYD1* complex (left) are less than that in the *apo* form (right). (**D**) The flexibility of DNA-binding loops in the complex (left) is less than that in the *apo* form (right). (**E**) The *B*-factors of the residues involved in hydrogen bonding in the swapped C/N terminus of the complex (left) are less than those in the *apo* PWWP domain (right). The residues involved in each compared region are presented as sticks.

A structural comparison between the *apo* PWWP domain and the PWWP-*SMYD1* complex reveals some major conformational differences, including alterations of the hinge loop region, changes in αA, the appearance of strand β5 and the distinct folding of monomers and dimers. The overall *B*-factor of the PWWP-*SMYD1* complex is much smaller than that of the *apo* form (Fig. 4A). We further compared the average *B*-factors of the regions with notable differences between two structures: the hinge loop; the DNA-binding region; αA and β5; the hydrogen-bonding residue pairs for dimerization of the complex and the corresponding residues of the monomeric *apo* form. To properly compare the *B-*factors between two structures, we normalised *B*-factor at the above-mentioned regions. The normalised *B*-factor for each region is a ratio calculated by dividing the average value for the region by the overall *B*-factor for the whole molecule. All normalised *B*-factor ratios of the regions involved in structural differences between the complex and the *apo* form are shown in Table S3.

For the largest structural differences at the hinge loop (Asp31 – Lys44) (Fig. 2F), the normalised *B*-factor of the newly formed helix αC is 1.0 at each chain in the PWWP-*SMYD1* complex. In contrast, the normalised *B*-factor of this same region is 1.51 in the *apo* PWWP domain (Fig. 4B), suggesting that the hinge loop region is relatively rigid in the PWWP-*SMYD1* complex but much flexible in the *apo* PWWP domain. This similar structural flexibility could be observed from the electron density map at this region of two structures (Fig. S3). The density map is broken from Asp31 to Lys44 in the *apo* PWWP domain (Fig. S3A), whereas the electron density is continuous in this region in the complex (Figs 2D and S3B). Next, the region from αA to β5 (Pro60 – Pro65) and the DNA-binding region, loop1 (Lys19 – Gly22) and loop4 (Asn77 – Lys80), are also relatively flexible in the *apo* form (Fig. 4C,D). Finally, the normalised *B*-factor (0.8) of the hydrogen-bonding residues for swapped dimerization of the PWWP-*SMYD1* complex is lower than that (0.94) of the corresponding residues of the monomeric *apo* PWWP domain (Fig. 4E). Taken together, the structure of the HDGF PWWP-*SMYD1* complex is more rigid and stable than that of the *apo* PWWP domain.

### *SMYD1* binding attenuates the interaction of HDGF with NCL

HDGF is known to be related to various cancers^6–8,10^. The colony formation assay, a well-used indication for cancer research^34^, was performed for confirming whether the recombinant HDGF and PWWP domain retains biological functions. The results showed that HDGF exhibited functional ability to promote colony formation better than the PWWP domain because of the dominant NLS2 in the C-terminus of HDGF (Fig. S4).

**Figure 5:**
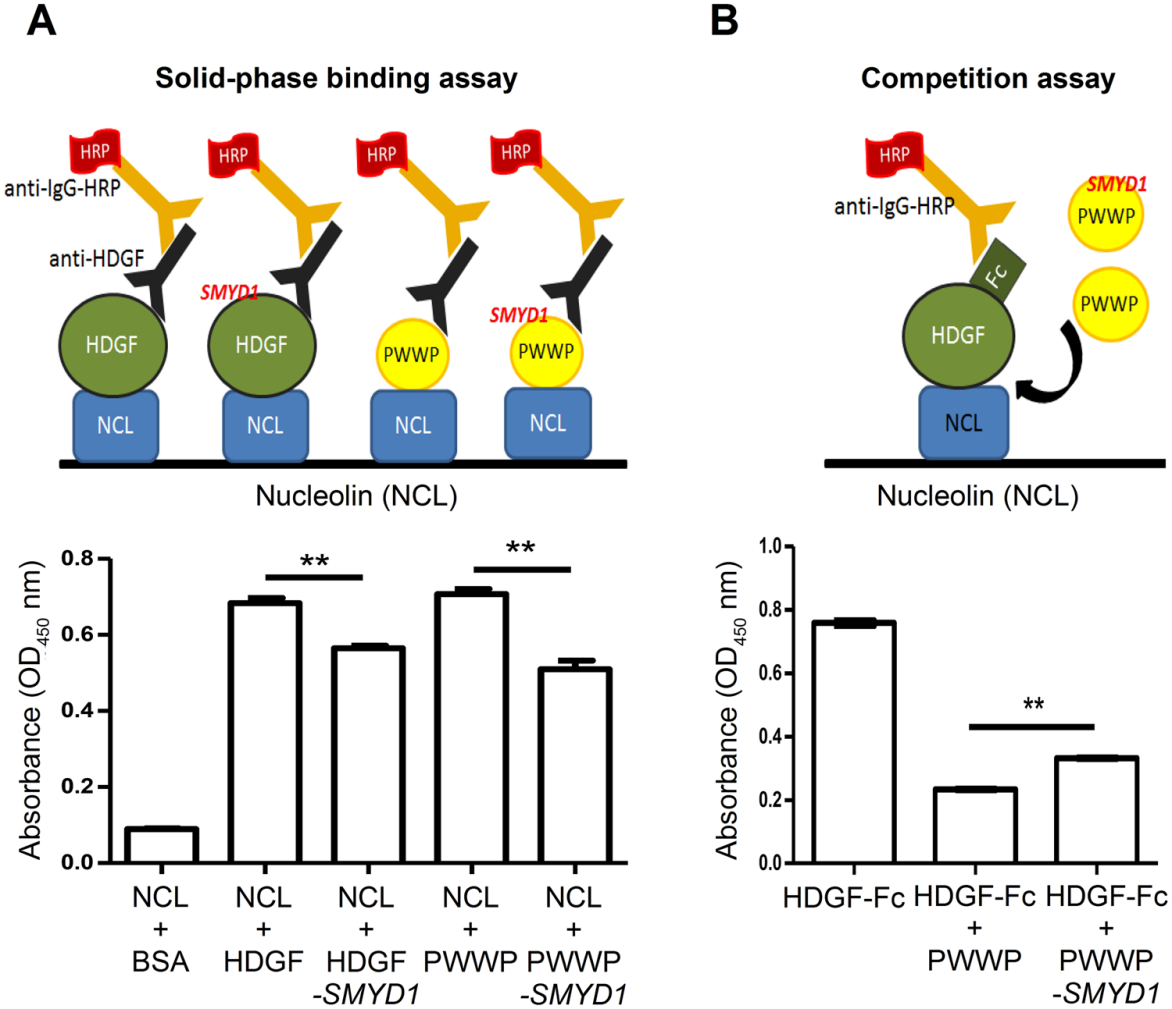
The NCL-binding capabilities of HDGF and the *apo* PWWP domain. (**A**) The solid-phase binding assay of the *apo* HDGF, the HDGF-*SMYD1* complex, the *apo* PWWP domain and the PWWP-*SMYD1* complex. The designed diagram is shown in the upper panel, whereas the solid-phase binding result is shown in the lower panel. The NCL-binding capability of both HDGF and the PWWP domain is attenuated by *SMYD1*. (**B**) The designed diagram showing the competition of the PWWP domain and HDGF-Fc with NCL is shown in the upper panel. The PWWP domain exhibits competition ability for the interaction between HDGF-Fc and NCL; however, the competition capability is reduced by *SMYD1*, suggesting that the PWWP domain is essential for the HDGF-NCL interaction (lower panel). Values that differ significantly from controls are indicated as **P < 0.01 by one-way ANOVA.

Moreover, it has recently been demonstrated that HDGF interacts with membrane nucleolin (NCL) via the PWWP domain^6^. In addition to the plasma membrane, such an interaction mainly occurs in the nucleus, where HDGF and NCL are abundant. Because HDGF also binds to *SMYD1* ^12^, we would like to delineate whether *SMYD1* binding affects the interaction between HDGF and NCL. It has been found that HDGF and the PWWP domain selectively bind to the NCL-coated matrix by the solid phase binding assay (Fig. 5A). Interestingly, the HDGF-*SMYD1* and PWWP-*SMYD1* complexes exhibited a significantly reduced binding capacity to NCL compared with unbound *apo* HDGF and the *apo* PWWP domain, respectively. Subsequently, we have employed the competition assay and have revealed that the excessive PWWP domain potently disrupts the interaction between HDGF and NCL, whereas the PWWP-*SMYD1* complex is less effective (Fig. 5B). Together, these results indicate that *SMYD1* binding interferes with the interaction of HDGF with NCL in the nucleus.

## Discussion

### The multiforms of the PWWP domain for DNA binding

According to our results, the protein concentration can affect the formation of multiple forms of the *apo* PWWP domain, and the dimeric *apo* PWWP domain can be readily obtained by increasing the protein concentration during purification (Fig. S1C, S1D). The *apo* PWWP domain has also been demonstrated to potentially form a dimer to bind to heparin on the cell surface with a binding affinity greater than that of the monomer; its transformation between dimers and monomers depends on the freshness of the sample preparation^27^. The dimeric and monomeric forms of the *apo* PWWP domain can hence co-exist, depending on the environment with variable conditions.

The PWWP domain is well known to be able to interact with the promoter and nonspecific DNA^12,31^. Our results show that the *apo* PWWP domain can form complexes with *SMYD1* of various lengths (Fig. 2A). Size calibration chromatography indicates that the *apo* PWWP domain and the PWWP-5 bp *SMYD1* complex exhibit as monomers, whereas the PWWP-10 bp and PWWP-15 bp *SMYD1* complexes exist as dimers. These observations might explain how the monomeric PWWP domain can still possess functions similar to dimers, such as heparin^28^ and DNA-binding abilities^31^ (Fig. 2A). It is thus reasonable to assume that the monomeric *apo* PWWP domain is capable of binding to the 5-bp *SMYD1* through its two intrinsic loops— loop1 (Lys19 – Gly22) and loop4 (Asn77 – Lys80) (Figs 2A and S5A). In contrast, the PWWP domain may utilize a dimeric structure to interact with DNA larger than 10 bp during its contact with DNA in the nucleosome under physiological conditions (Fig. 2A,B).

Correspondingly, structural comparison of the HDGF PWWP-*SMYD1* complex and the *apo* PWWP domain reveals several significant conformational distinctions (Fig. 2F). The complex stretches its overall structure into an extended shape as a domain-swapped dimer from the globular shape of the *apo* form in the monomer. The distance between two DNA fibres in a nucleosome is ~5.5 nm, which is about the dimension (~6 nm) of the extended main-chain structure of the PWWP-*SMYD1* complex, suggesting that the PWWP domain may explore the methylated histone and the promoter with both swapped C/N and N/C termini^35^.

Based on domain swapping to exchange the similar domains between two protein molecules (Fig. S5A), the pattern of hydrogen-bonding residue pairs of one swapped terminus in the PWWP-*SMYD1* complex is similar to that in internal interactions of the monomeric *apo* PWWP domain (Fig. S5B). The amount and average bond length of these hydrogen-bond pairs are, however, different in the two forms. This condition thus implies that fewer hydrogen bonds (9 *vs*. 13) with a larger bond distance in the *apo* PWWP domain may be just sufficient to maintain the globular structure of a monomer with larger temperature factors, and provide the possibility for domain swapping to form a much more stable dimer of the complex (Fig. 2F and Table S1 and S4).

### Dimerization by domain swapping of the HDGF PWWP domain for DNA binding

**Figure 6:**
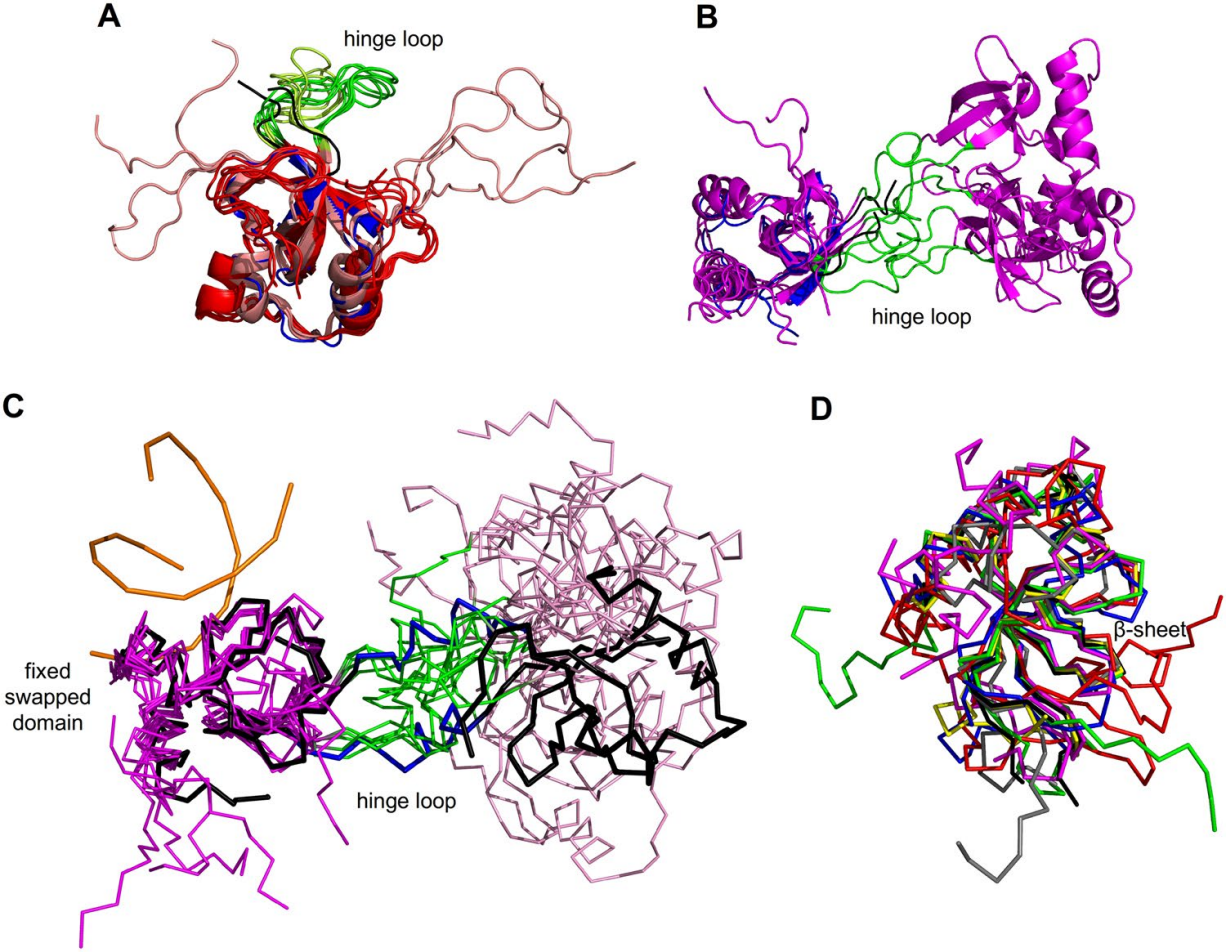
Structural comparison of various PWWP domains. (**A**) The crystal structure of the *apo* PWWP domain (blue) and the NMR solution structure (PDB entry: 1RI0, human HDGF PWWP domain; red) and (PDB entry: 2B8A, mouse HDGF PWWP domain; salmon) comprise a flexible hinge loop, loop2, which is shown in black for the crystal structure and green and yellow for the NMR solution structure. (**B**) The hinge loop is flexible in the NMR structure of the dimeric *apo* PWWP domain with multiple conformations (PDB entry: 2NLU, magenta), beginning from the hinge loops shown in green, similar to the crystal structure (blue and black). (**C**) Structural comparison of the dimeric PWWP-*SMYD1* complex (black) and the dimeric *apo* PWWP domain (magenta). Alignment of one swapped domain shows that the other swapped domain of the PWWP-*SMYD1* complex exhibits a stable conformation and a rigid domain orientation because of the newly formed αC (blue) transformed from the hinge loop, differing from the dimeric *apo* PWWP domain (pink) with variable orientations beginning from the hinge loop (green). (**D**) The main structural features of various PWWP domains (mouse HDGF, PDB: 28BA, green; MSH6, 2GFU, red; LEDGF/p75, 2M16, blue; human HDGF, 2NLU, gray; HDGF2, 3EAE, yellow; DNMT3B, 5CIU, magenta; human HDGF, 5XSL, black) are similar with the conserved β-barrel region and α-bundles in the C-terminus.

The intrinsic flexibility of the hinge loop with partial density of the *apo* PWWP domain is consistent with multiple conformations of loop2 in the NMR solution structure (PDB entries: 2B8A, 1RI0, 2NLU) (Fig. 6A,B). The refolded dimeric PWWP domain without DNA in the NMR structure (PDB entry: 2NLU) exhibits domain swapping with the flexible loop2 (Glu32 – Lys44) under an ultrahigh concentration (100 mg/mL). In our PWWP-DNA complex structure, however, the flexible loop alters its conformation into an α-helix. Moreover, the structural orientations of the *apo* dimeric PWWP domain obtained with NMR and the dimeric PWWP-*SMYD1* complex with X-ray are variable, beginning with its flexible loop2 upon superimposing these two structures on one swapped terminus (Fig. 6C). The flexible loop2 in the *apo* form is transformed to a rigid helix αC in the PWWP-*SMYD1* complex, indicating that loop2 may be involved in the domain swapping. In some domain-swapped proteins, the transformation of a hinge loop to an α-helix or β-strand indicates that the dimer or oligomer form is favoured over the monomer^33,36^. The energy barrier for domain swapping of oligomers could be reduced under several conditions, such as temperature changes, protein mutations, increased protein concentrations and the binding of a ligand^33^. The PWWP domain should hence be stable with a swapped dimeric form to interact with DNA more than with a monomeric form. The domain-swapped PWWP domain thus increases larger DNA-binding surfaces, which may benefit the DNA interactions.

The structure of the *apo* PWWP domain shows an interpretable electron density but with an overall *B*-factor that is larger than for the complex (Fig. 4A). It is thus reasonable to assume that the activation energy of the flexible monomeric PWWP domain may be decreased upon altering its conformation to form a more rigid dimer during attachment to DNA. Additionally, the *B*-factor values of the relevant residues that participate in protein dimerization and DNA interactions in the PWWP-*SMYD1* complex are much smaller than those in the *apo* PWWP domain. These observations indicate that DNA might help not only to alter the conformation of the hinge loop in the PWWP domain but also to stabilize the complex conformation (Fig. 4A and Table S3).

The overall secondary-structure content of HDGF contains a large fraction of random coils as shown by SRCD, whereas the structure of the *apo* PWWP domain comprises mostly β-strands, indicating that the C-terminus of HDGF may be flexible with a large portion of the coil structures (Figs 1A and S6). The previous work has shown that a protein containing a large portion of random coils can undergo domain swapping without interference^37^. The coiled C-terminus of HDGF hence may not affect domain swapping of the N-terminal PWWP domain as observed in the structure of the PWWP-*SMYD1* complex.

### Structural comparison with other PWWP domains and implications

A structural comparison of the *apo* HDGF PWWP domain with other PWWP domains from mouse and human HDGF, HDGF2, mismatch repair protein MSH6, LEDGF/p75, DNMT3B (PDB entries: 2B8A, 2NLU, 3EAE, 2GFU, 2M16 and 5CIU) shows that these PWWP domains comprise four or five stranded β-barrel and α-helices in their C-terminus, indicating that the main structural features of those PWWP domains are conserved, especially for the β-barrel region (Fig. 6D).

Before our work, no structure of the PWWP-*SMYD1* complex was available, but several research groups had attempted to map the DNA-binding region with NMR chemical-shift perturbation experiments^31,32,38^. Previous studies of the DNA-binding model of the mouse HDGF PWWP domain (PDB entry: 2B8A) indicated that the N-terminus (Lys8 – Tyr10), the loop region (Lys19 – Tyr23) and two α-helix regions (Gly59 – Leu63; Tyr66 – Phe73), which contribute to the positively charged surface, were involved in DNA binding, especially with Glu9, Tyr10, Gly22, Lys61 and Arg79^31^. Based on our crystal structure of the PWWP-*SMYD1* complex, the DNA-binding regions are loop1 (Lys19 – Gly22), which is before the highly conserved PWWP motif, and a partial loop4 (Asn77 – Lys80), differing from the prediction obtained with NMR chemical-shift perturbation. This result potentially suggests that the PWWP domain interacts with a longer region of DNA through larger regions (Fig. 3 and S7A).

The identified interactions between two DNA-binding loops and the minor groove of DNA, which comprises ~ four base pairs with an axial dimension ~12 Å in the *B*-form DNA, may explain the obvious chemical shift of interactions between the PWWP domain and the 15-bp DNA detected only in the middle 5 bp of DNA^31^ (Fig. S2). Both NMR chemical-shift perturbation analyses of the PWWP domain of Human Mismatch Repair Protein -MSH6 (PDB entry: 2GFU) with 35-bp DNA and the PWWP domain on the Lens epithelium-derived growth factor (LEDGF) (PDB entry: 2M16) with DNA suggested that some residues involved in DNA binding occur in the loop1 region and the residues of the corresponding loop4 region^32,40^ (Fig. S7A). Our human HDGF PWWP-*SMYD1* complex further provides direct evidence and interaction details of loop1 (Lys19 – Gly22) and loop4 (Asn77 – Lys80) with DNA in the PWWP domain (Fig. S7A). In addition, the PWWP domain can interact with a longer region of DNA through its basic patch^32,40^.

In our HDGF PWWP-*SMYD1* complex, the PWWP domain contacts DNA with residues of varied types that are surrounded by a positively charged area, especially loop1 and loop4. The sequence alignment of various PWWP domains shows that the DNA-binding loop1 contains highly conserved residues, whereas loop4 comprises only partially conserved residues. The sequences _77_NKRK_80_ of loop4 in the isoforms of HDGF, HDGF2 and HDGF3 are, however, identical, indicating that all isoforms of HDGFs may also interact with DNA via this loop region (Figs 3A and S7A).

From a structural perspective, the DNA-binding residues in the HDGF PWWP-*SMYD1* complex are located on loop1 and loop4, which are within the proposed DNA-binding area in those structures monitored by NMR chemical shift perturbation (PDB entries: 2B8A, 2M16, 2GFU), implying that these PWWP domains may specifically contact DNA through the identified residues at the two loop regions (Fig. S7B). The orientation of loop4 in the MSH6 PWWP domain is different from other PWWP domains. Although the flexibility of this loop from all NMR models of the MSH6 PWWP domain is relatively rigid, it may also alter its orientation when interacting with DNA.

### The importance of the positively charged distribution in the complex

Previous work using the electrophoresis mobility shift assay (EMSA) to examine truncated HDGF with 80-bp *SMYD1* showed that the truncated proteins HDGF_1-35_, HDGF_1-70_, HDGF_70-237_ cannot interact with 80-bp *SMYD1*, whereas HDGF_35-237_ retains a partial binding ability with 80-bp *SMYD1* ^12^. Assuming no severe influence on protein folding by truncation, all of those truncated regions lack one of two DNA-binding loops, either loop1_19-22_ or loop4_77-80_. As mentioned, the PWWP domain is equipped with a positively charged surface on only one side of the molecular surface (Fig. 1E). We found that the region from Lys39 to Lys80 contributes to a positively charged surface, which is included in the _35_HDGF_237_ (Figs 1E and 3B). In addition, Lys8, Lys19 – His25, Lys39 – Lys44, Leu58 – Phe64 and Lys72 – Lys80 compose the basic patch in the PWWP domain, which may be involved in interactions with the longer DNA. We therefore reasonably assume that DNA should be first recruited by the positively charged force on one side of the exposed surface and subsequently interacts with the DNA-binding regions, which is identified in this work, in the PWWP domain.

### Hypothesis of the mechanism of DNA binding and the functional process

**Figure 7:**
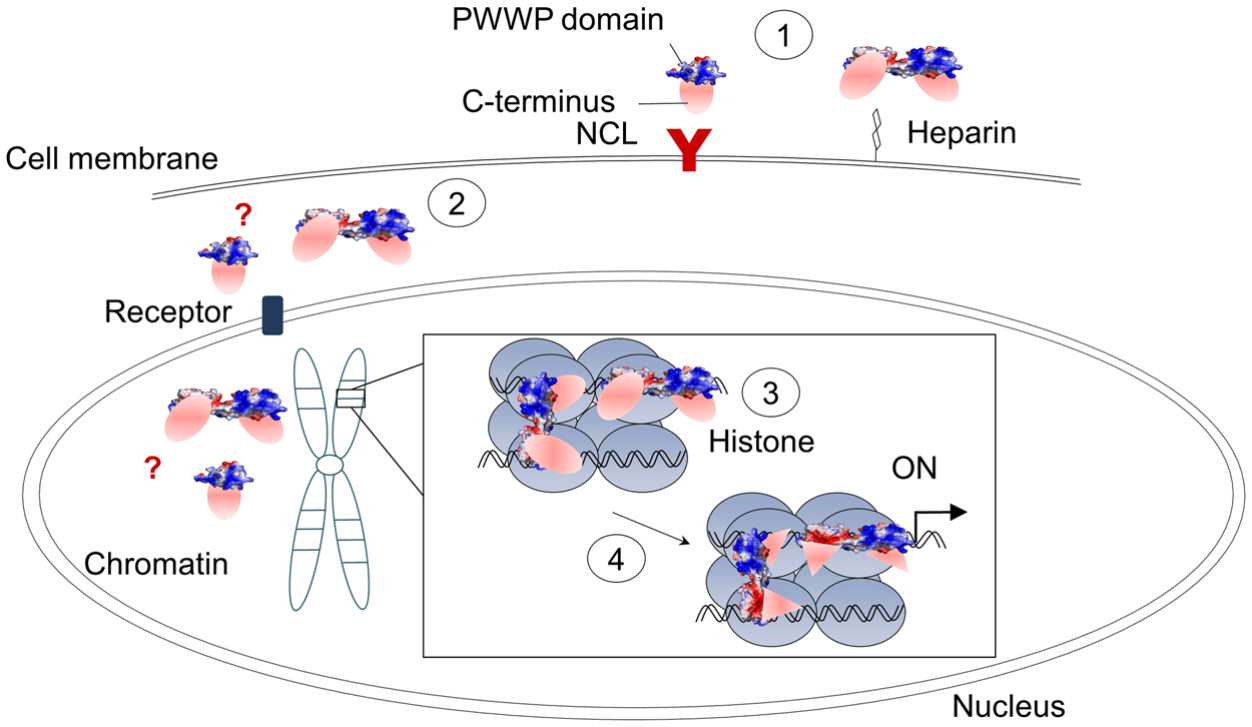
Hypothesis for the role of HDGF in gene regulation. The HDGF molecule comprises an N-terminal PWWP domain and a C-terminus (red oval). Step 1, At a higher concentration, HDGF may interact with heparin on the cell surface with an enhanced binding affinity via its dimeric structure or bind to heparin with a monomeric form with a low affinity, or alternatively bind to the membrane receptor, NCL. Step 2, Dimeric or monomeric HDGF recognizes the receptor on the nuclear pole with its specific sequence of NLS2 in the variable C-terminus to translocate HDGF into the nucleus after internalization into the cell. Step 3, Either monomers or dimers of HDGF might exist inside the nucleus. The HDGF undergoes domain swapping to form a rigid dimer linked with a flexible hinge loop, which is transformed into a helix αC when approaching the histone or DNA fibres, as shown in the enlarged box. HDGF might lie across two DNA fibres because the structural dimension of a dimer is larger than the distance between two DNA fibres, or it might slide over one DNA fibre through its highly conserved PWWP domain. Step 4, After the PWWP domain is activated by methyl peptides in histone and recognizes the promoter, HDGF might alter its C-terminal conformation (red triangle) and participate in regulating the gene expression through its variable C-terminus.

Taken together, we propose a hypothesis for the mechanism of DNA binding and the functional process of HDGF in gene regulation (Fig. 7). The growth factor HDGF is expressed in the highly developed foetal tissue, and over-expressed HDGF causes a greater risk of various cancers^4,39,40^. Hence, HDGF might first interact with heparin on the cell surface with its dimeric and monomeric forms with distinct binding affinities^27^, or bind to NCL, a plasma membrane receptor of HDGF^6^. After internalizing into the cell, the HDGF recognizes the receptor on the nucleus pole by its specific sequence of NLS2 in the variable C-terminus to translocate itself into the nucleus^14^. The interactions of monomeric or dimeric HDGF with the receptor remain unclear. Subsequently, HDGF of dimers or monomers may gather together in the nucleus under physiological conditions. Next, HDGF may undergo domain swapping with the PWWP domain, and the swapped dimeric HDGF in the nucleus transforms its flexible hinge loop further into a rigid hinge α-helix when the protein approaches DNA fibres with loop1 and loop4. Once the PWWP domain is activated by methyl peptides of histone and recognizes the promoter, the synergistic binding mechanism may enhance the binding affinity with the corresponding histone and DNA and participate further in the regulation of gene expression through the C-terminus of HDGF^12,15,30^.

In summary, we have determined the first crystal structures of the HDGF PWWP domain in complex with *SMYD1* of 10 bp and its *apo* form, respectively. The PWWP domain undergoes domain swapping to markedly transform its secondary structures and to alter the overall conformation from monomeric globular folding into an extended dimeric structure upon binding to DNA. The PWWP-DNA complex becomes more rigid and stable than the *apo* form. The PWWP domain utilizes residues with variable characters at loop1 and loop4 from two chains of the swapped dimer to interact with the minor grooves of DNA. Together with physiological assays, these novel structural findings provide new insights into the mechanism of DNA binding and the functional process of HDGF.

## Methods

### Construction of the HDGF PWWP_1-100_ domain

The construct of human HDGF was amplified from the library of human fetal brain cDNA (Stratagene, La Jolla, CA) utilizing the polymerase chain reaction (PCR) as described previously^41^. After the DNA sequencing analysis, the PCR-amplified PWWP domain was subcloned into *NdeI* and *EcoRI* sites of the pET28a vector and transformed into *Escherichia coli* (*E. coli*) BL21-Codon Plus^®^-*RIL* for expression and purification of the recombinant PWWP protein. All constructed plasmids were confirmed by DNA sequencing.

### Expression and purification of the HDGF PWWP_1-100_ domain from *E. coli*

A single colony of the bacteria was selected and placed into Luria-Bertani (LB) broth (20 mL) with kanamycin (50 μg/mL) at 37 °C overnight. The culture was then transferred into fresh LB medium (2 L) with kanamycin (50 μg/mL). After the OD_600_ reached 0.4–0.6, isopropyl β-D-thiogalactopyranoside (IPTG, final concentration 1 mM) was added to the culture for induction with rotary shaking at 37 °C for another 20 h. The cells were harvested by centrifugation (6,000 × *g*) at 4 °C for 10 min.

The cells were resuspended in lysis buffer (20 mM Tris-HCl, pH 7.5) and then sonicated by ultrasonication (SONICS VCX 750) using a pulsation cycle (2 s on, 4 s off) for a total duration of 12 min of sonication at 40% energy on ice. The soluble PWWP protein extract was collected by centrifugation (12,000 × *g*) at 4 °C for 30 min; the supernatant was loaded onto a Ni^2+^-NTA agarose column (GE healthcare) that had been pre-equilibrated with binding buffer (20 mM Tris-HCl, pH 7.5). The unwanted proteins were washed out with binding buffer containing imidazole (20–50 mM); the desired target protein of the PWWP domain was eluted with binding buffer containing imidazole (300 mM). The protein was further purified by anion-exchange chromatography (Hitrap Q) with buffer A (20 mM Tris-HCl, 5 mM EDTA, 5 mM β-ME pH 7.5) and buffer B (20 mM Tris-HCl, 1 M NaCl, 5 mM EDTA, 5 mM β-ME pH 7.5) while monitoring the ratio of A260/280 based on the markedly distinct p*I* values of the *apo* PWWP domain and DNA during purification. This purification step removed endogenous DNA from *E. coli* and contaminated proteins to ensure that there was no interference and contamination of endogenous DNA for further structural studies and physiological assays^28,32^. The homogeneity of the PWWP domain protein was confirmed by size-exclusion chromatography (Superdex-200) with buffer (20 mM Tris-HCl, 5 mM EDTA, 5 mM β-ME, pH 7.5). The ratio of A260/280 was examined, and showed that the purified *apo* PWWP domain was not contaminated with endogenous DNA from *E. coli*. The PWWP protein was concentrated (on Centricon, MWCO 10,000; Satorius Vivapin 20) before crystallization. The purity of the final PWWP domain was greater than 95% based on SDS-PAGE (15%) and staining (Coomassie Brilliant Blue R-250).

### Expression and purification of HDGF from *E. coli*

The expression and purification procedures for full-length HDGF were similar to that for the *apo* PWWP domain described in the above section. A pre-equilibrated binding buffer (20 mM Tris-HCl, 150 mM NaCl, pH 7.5) was prepared for a Ni^2+^-NTA agarose column. The unwanted proteins were washed out with binding buffer containing imidazole (20–50 mM), and the desired target protein of HDGF was eluted with a binding buffer containing imidazole (150 mM). The subsequent step with anion-exchange chromatography (Hitrap Q) was similar to that for the *apo* PWWP domain. The homogeneity of the full-length HDGF protein was confirmed by size-exclusion chromatography (Superdex-200) with buffer (20 mM Tris-HCl, pH 7.5, 150 mM NaCl, 5 mM EDTA, 5 mM β-ME). HDGF was concentrated (on Centricon, MWCO 10,000; Satorius Vivapin 20) before colony-formation and Western-blot assays and structural studies. The purity of HDGF was greater than 95% based on SDS-PAGE (12%) and staining (Coomassie Brilliant Blue R-250).

### Purification of the HDGF PWWP-*SMYD1* complex

After purification by the ion-exchange chromatography to exclude endogenous DNA from *E. coli*, if any, we incubated DNA of various lengths with the sequence of the *SMYD1* (15, 10 and 5 bp) and the PWWP domain at 4 °C overnight. The *SMYD1* sequences were 5′-CAGGCTGGTCTTGAA-3′, 5′-CAGGCTGGTC-3′ and 5′-CAGGC-3, respectively. Complementary single-stranded DNA molecules ordered from the commercial company (*Mission Biotech*) were mixed together and then heated at 95 °C for 10 min. After heating, the DNA mixtures were left near 23 °C overnight to form the double-stranded DNA. The monomeric PWWP domain and *SMYD1* were incubated at a molar ratio of 1:1. The complex of the PWWP domain and *SMYD1* was purified again by size-exclusion chromatography (Superdex-200) in a buffer (20 mM Tris-HCl, 5 mM EDTA, 5 mM β-ME, pH 7.5) to ensure that the PWWP-*SMYD1* complex of high homogeneity was properly collected and that unbound DNA was removed to avoid a perturbing effect on the subsequent crystallization.

### Solid-phase binding assay

The solid-phase binding assay was performed to detect the binding ability of HDGF, HDGF-15 bp *SMYD1* complex, the PWWP domain and the PWWP-15 bp *SMYD1* complex to the nucleolin (NCL) protein. Human NCL cDNA was constructed using the pET15b vector and transformed into *E. coli* BL21 (DE3) cells. After IPTG (1 mM) induction, the protein was purified using the Ni^2+^-NTA agarose column. For the solid-phase binding assay, NCL coating was applied at 10 µg/mL at 4 °C overnight. Each well was blocked with BSA (3%) in PBS buffer containing Tween-20 (0.05%) at 25 °C for 1.5 h. Antigen proteins (HDGF and PWWP) were reacted at 25 °C for 1.5 h. The PWWP-specific antibody and the HRP-conjugated antibody were used to detect HDGF and PWWP domain proteins. Each well was reacted in TMB substrate at 25 °C, and H_2_SO_4_ (1 N) was applied as a stop solution. Finally, the optical density of each well was measured at 450 nm using an ELISA reader.

### Competitive assay

The competitive assay was performed to validate whether the PWWP domain was competitive for the HDGF-NCL interaction. For this assay, the human HDGF cDNA was constructed in the pcDNA3.1 expression vector using a fusion of the human antibody-Fc fragment. For protein production, the plasmid was transfected into human embryonic kidney 293 T cells using Lipofectamine^®^ 2000 reagent. The conditional medium was collected, and the HDGF-Fc protein was purified using a protein G Sepharose^®^ column. In the competitive assay plate, NCL was coated at a concentration 10 µg/mL at 4 °C overnight. After BSA (3%) blocking, the HDGF-Fc fusion protein (0.01 μM) was reacted at ten times the molar concentration of the PWWP domain (0.1 μM) and PWWP*-SMYD1* (0.1 μM) for competition. The goat anti-human-HRP antibody was used for detection of the HDGF-Fc fusion protein. Finally, the TMB substrate and H_2_SO_4_ (1 N) as a stop solution were used for the colour reaction.

### Crystallization of the *apo* PWWP domain and HDGF PWWP-*SMYD1* complex

The purified PWWP domain and HDGF PWWP-*SMYD1* complex proteins were concentrated to 10–20 mg/mL in a storage buffer (20 mM Tris-HCl, 5 mM EDTA, 5 mM β-ME, pH 7.5) containing protease inhibitor cocktail (1%, Roche). Crystallization trials were performed in 96-well plates (JET Biofil) using several crystal-screening kits based on the hanging-drop vapour-diffusion method utilizing hanging drops (0.4 μL) containing equal volumes of protein solution (0.2 μL) and a reservoir solution (0.2 μL) against the bottom reservoir solution (100 μL) at 8 and 18 °C, respectively. The *apo* PWWP in storage buffer with spermine tetrahydrochloride (10 mM), which functioned as a kind of precipitant for DNA to remove soluble DNA, was crystallized under conditions containing sodium chloride (200 mM), Tris (100 mM, pH 8.5), and polyethylene glycol (PEG) 4000 (25%, *w*/*v*). The PWWP-*SMYD1* complex was crystallized under conditions containing sodium nitrate (90 mM), sodium phosphate dibasic (90 mM), Tris; Bicine (100 mM, pH 8.5), PEG 1000 (12.5%) and PEG 4000 (12.5%, *w*/*v*).

### X-ray data collection, structure determination and refinement

The crystals were mounted on a synthetic nylon loop (0.05–0.1 mm, Hampton Research Co.) and then flash-cooled in liquid nitrogen. The protein crystals were first tested with X-rays from synchrotrons at TLS beamlines BL13B1, BL13C1 and BL15A1 at the National Synchrotron Radiation Research Center (NSRRC) in Taiwan and BL12B2 at SPring-8 in Japan. For complete data collection of the *apo* PWWP domain at the best resolution, a total rotation of 120° with oscillation of 1.0° were measured at BL44XU of SPring-8 using an X-ray wavelength of 0.9 Å with an exposure duration of 1 s and distance of 500 mm from the crystal to the detector at 110 K under a nitrogen stream provided by a cryo-system (X-Stream, Rigaku/MSC, Inc.). For complete data collection of the PWWP-*SMYD1* complex, a total rotation of 200° with oscillation of 0.5° were measured at the Taiwan Photon Source (TPS) beamline 05A equipped with a CCD detector (MX300-HS, Rayonix) using an X-ray wavelength of 1.0 Å, exposure duration of 0.1 s and distance of 350 mm from the crystal to the detector at 110 K under a nitrogen stream provided by a cryo-system. All data were indexed, integrated and scaled using the programme suite *HKL-2000* ^42^.

The crystal of the *apo* PWWP domain belonged to the space group *P*6_4_22, with unit cell dimensions *a* = b = 79.49 Å and *c* = 105.10 Å, and diffracted to 3.3 Å resolution. The crystal of the PWWP-*SYMD1* complex belonged to the space group *P*3_1_, with unit-cell dimensions *a* = *b* = 32.40 Å and *c* = 205.82 Å, and diffracted to 2.84 Å resolution. Structures of both the *apo* PWWP domain and the PWWP*-SMYD1* complex were determined using the molecular replacement method with the *Molrep* programme^43^. The crystal structure of the *apo* PWWP domain was first determined with the X-ray structure of the PWWP domain of human HDGF2 (PDB entry: 3QBY) as a search model. The HDGF PWWP-*SMYD1* complex was later determined with the solved structure of the HDGF *apo* PWWP domain as the protein model template and the structure of a 10-bp DNA as the DNA search model, respectively. All structures were built with *COOT* ^44^. Refinement was performed with *REFMAC5* and *CCP4* ^45^. The correctness of the stereochemistry of each structure was validated with *MolProbity* ^46^. The calculations of root-mean-square deviation from angles, bonds and dihedral and improper angles showed the satisfactory stereochemistry. All details of the crystallographic data and refinement statistics are shown in Table 1. The figures were prepared in *PyMOL* (http://www.pymol.org/).

### Accession codes

Coordinates and structure factors of the HDGF PWWP-*SYMD1* complex and the *apo* PWWP domain have been deposited in PDB under the accession codes 5XSK and 5XSL, respectively.

## Electronic supplementary material

Supplementary Information
