# Supplementary material for: Domain swapping and SMYD1 interactions with the PWWP domain of human hepatoma-derived growth factor
Source: Sci Rep. 2018 Jan 10;8:287. doi: 10.1038/s41598-017-18510-8 (PMC5762634; doi:10.1038/s41598-017-18510-8)
Supplement: Supplementary file 1 — Supplementary Information [file 41598_2017_18510_MOESM1_ESM.pdf]

Supplementary Information for

Domain swapping and *SMYD1* interactions with the PWWP domain of human hepatoma-derived growth factor

Li-Ying Chen, Yen-Chieh Huang, Shih-Tsung Huang, Yin-Cheng Hsieh, Hong-Hsiang Guan, Nai-Chi Chen, Phimonphan Chuankhayan, Masato Yoshimura, Ming-Hong Tai and Chun-Jung Chen

This PDF file includes:

Supplementary Methods: 1-2
Supplementary Figures: 1-7
Supplementary Tables: 1-4

Supplementary Methods

1. Colony formation assay

In the assay of the colony formation, SK-Hep1 cells were seeded with 1 x 10^3 cells per well in the 6-well plate and fed with DMEM medium containing calf serum (10%). Post cell adhesion, recombinant HDGF (10 ng/mL) and the PWWP domain (100 ng/mL) were added in DMEM medium containing calf serum (1%). Cells were cultured at 37 °C in humidified atmosphere with CO_2 (5%), and the fresh medium and recombinant proteins were replaced every three days. At the tenth day, culture plates were fixed with paraformaldehyde (4%), and then stained with crystal violet. Images of the colonies were captured, and colony numbers were counted with the statistical analysis.

2. Secondary structure analyses of the *apo* PWWP domain and HDGF

Circular-dichroism (SRCD) experiments were performed using a nitrogen-flushed instrument with X-rays from a synchrotron at TLS beamlines BL04B and BL04C at NSRRC. Samples were prepared in Tris buffer (20 mM, pH 7.5) before experiments. SRCD spectra were recorded near 23 °C for HDGF and the PWWP domain with concentrations 6 and 3 mg/mL, respectively, and the sample volume 20 µL. The measurements were repeated with three scans. The spectrum of background buffer was subtracted from the experimental data.

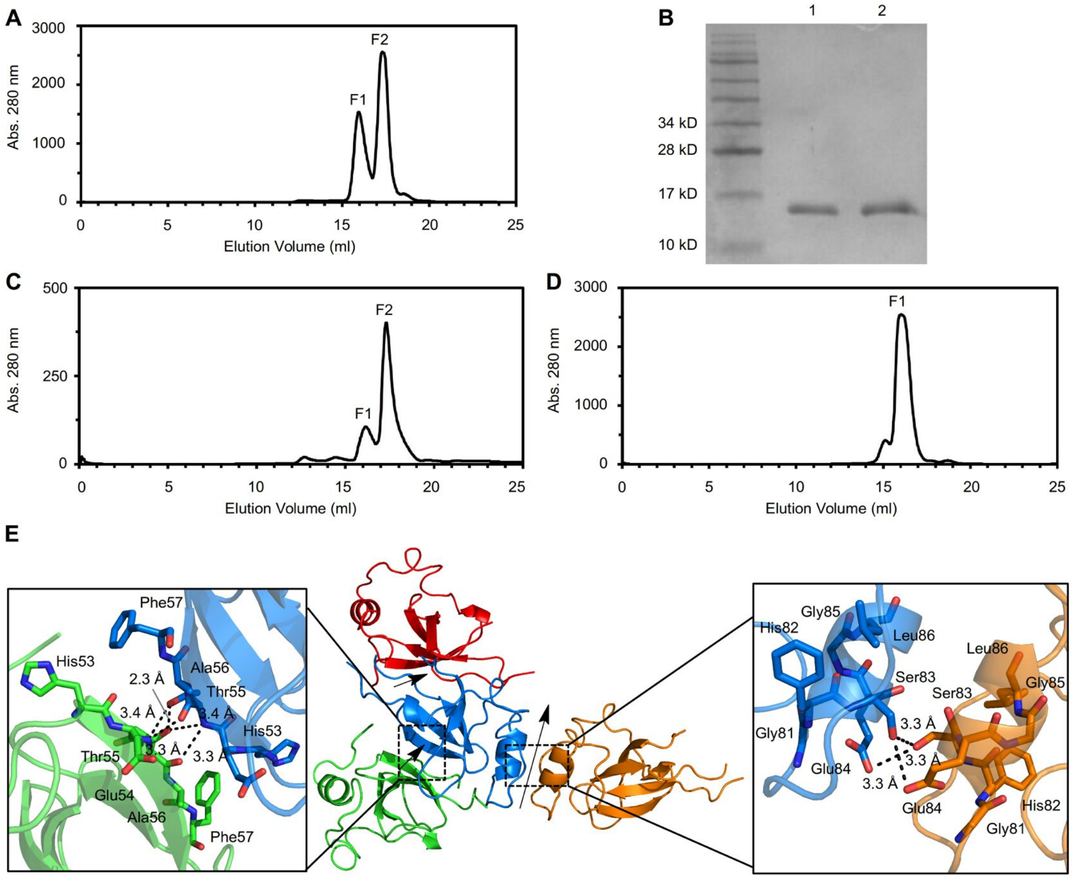

Figure S1. Observation of multiple forms of the PWWP domain with the size-exclusion chromatography. (**A**) Multiple forms of the PWWP domain were eluted at 16 mL (F1: dimer) and 17.5 mL (F2: monomer) with size-exclusion chromatography (Superdex-200). (**B**) The protein fractions were confirmed with SDS-PAGE. Lane 1: dimer; lane 2: monomer. The molecular mass of the PWWP domain is ~ 15 kDa. (**C**) Dominant monomeric PWWP domain was obtained at a low concentration (< 1.5 mg/mL). Also, the dimeric PWWP domain would change to monomers in buffer containing ionic-strength salts, such as NaCl (150 mM) within three days (**D**) The dimeric PWWP domain was acquired at a high protein concentration (> 1.5 mg/mL). (**E**) The symmetry-related *apo* PWWP domains in the crystals of hexagonal space group $P6_422$. The monomeric PWWP domain is shown in blue, whereas other symmetrically generated PWWP domain molecules related by crystallographic two-fold axes (arrows) are shown as green, red and orange, respectively. The detail contacts are presented in enlarged boxes.

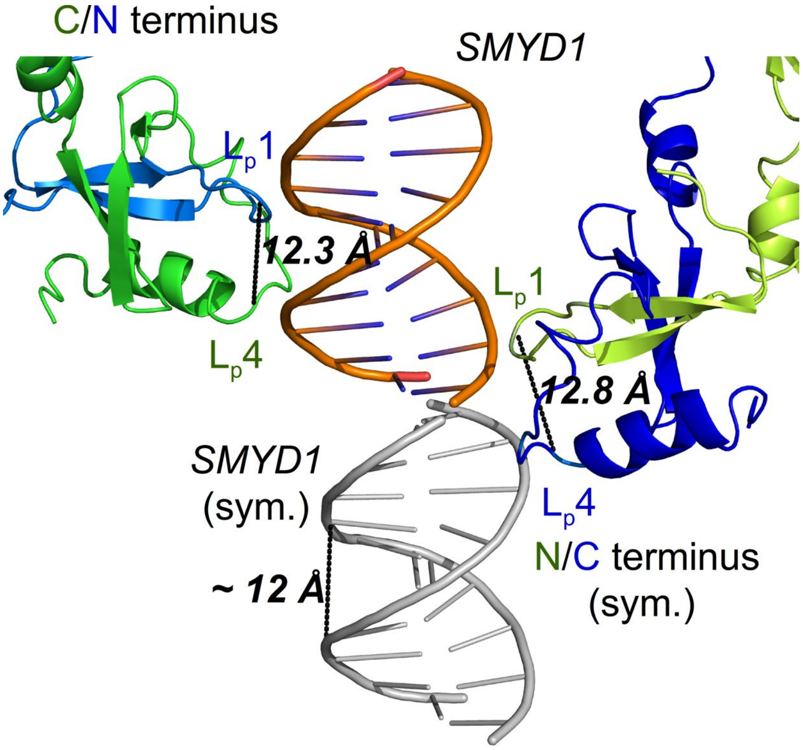

Figure S2. Distances between loop1 and loop4 in the PWWP-*SMYD1* complex. The distances (12.3 and 12.8 Å, both are from the N atom at Gly22 to the N atom at Lys80) between loop1 and loop4 from the C/N (green and marine) and the N/C termini (limon and blue; generated with the crystallographic symmetry of another complex) are near the spatial dimension (~ 12 Å) of the minor groove. Green and marine represent chains A and B, respectively. DNA molecules are shown in orange for the complex structure and gray for symmetry.

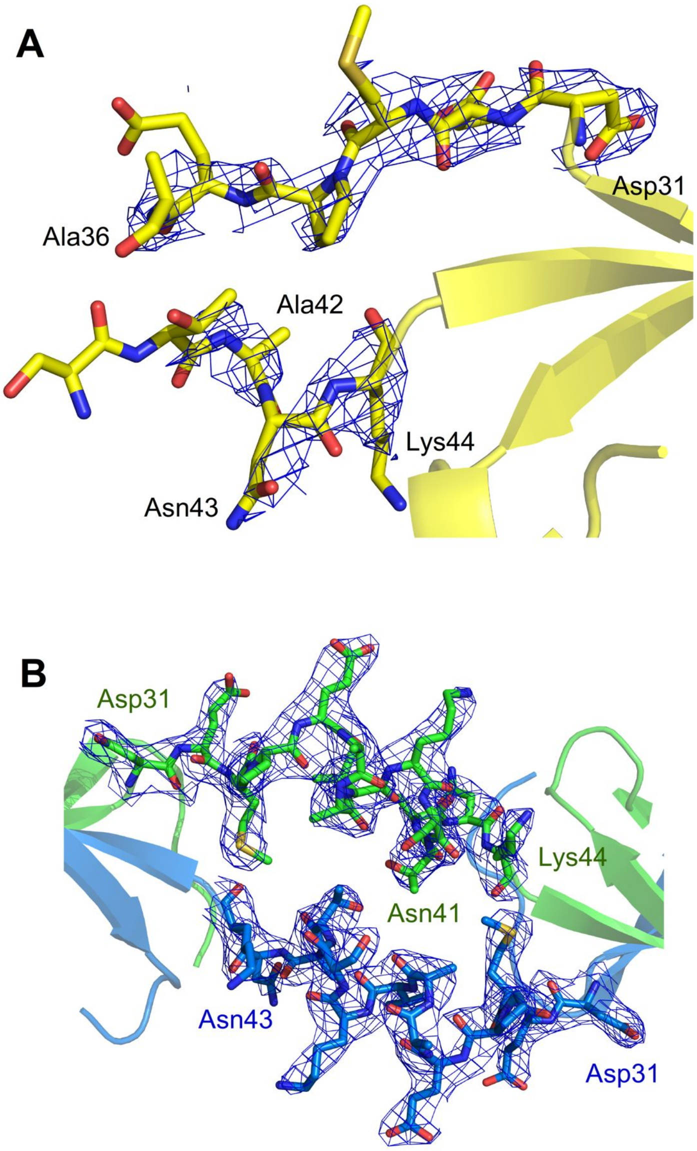

Figure S3. The flexibility of the hinge loop (αC) in the *apo* PWWP domain and PWWP-*SMYD1* complex based on electron density maps. (**A**) The structure is difficult for tracing by the incomplete density from Ala36 to Asn43 under $2F_o$-F_c at 1.5 σ (blue mesh). (**B**) The structure could be well determined and defined with the continuous density at the αC region from Asp31 to Lys44 (blue mesh, $2F_o$-F_c at 1.5 σ).

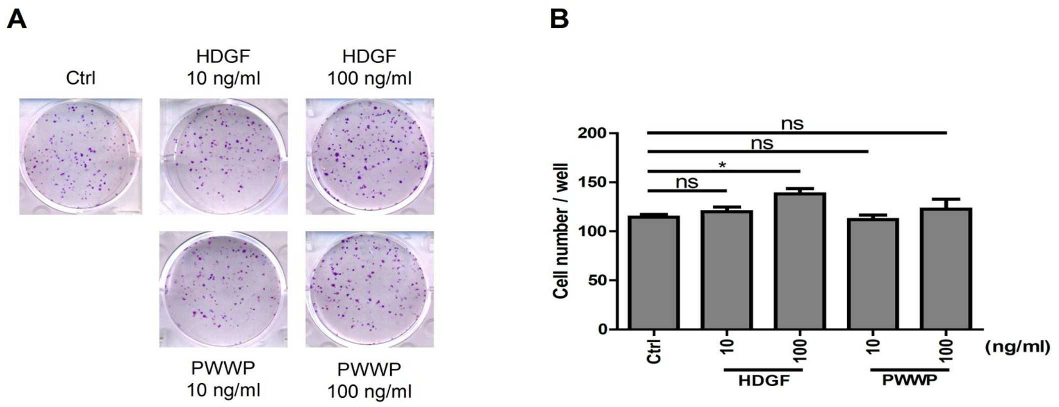

Figure S4. The biological activities of human HDGF and the *apo* PWWP domain. (**A**,**B**) The assays of the colony formation with HDGF and the *apo* PWWP domain. Treatments with HDGF (100 ng/mL) significantly enhanced the colony number of SK-Hep1 cells as compared with the control. In the treatment group with the *apo* PWWP domain, the colony numbers of cells were not notably changed. Values that differ significantly from controls are indicated as $^{*}P < 0.05$ by one-way ANOVA. ns: non-significant.

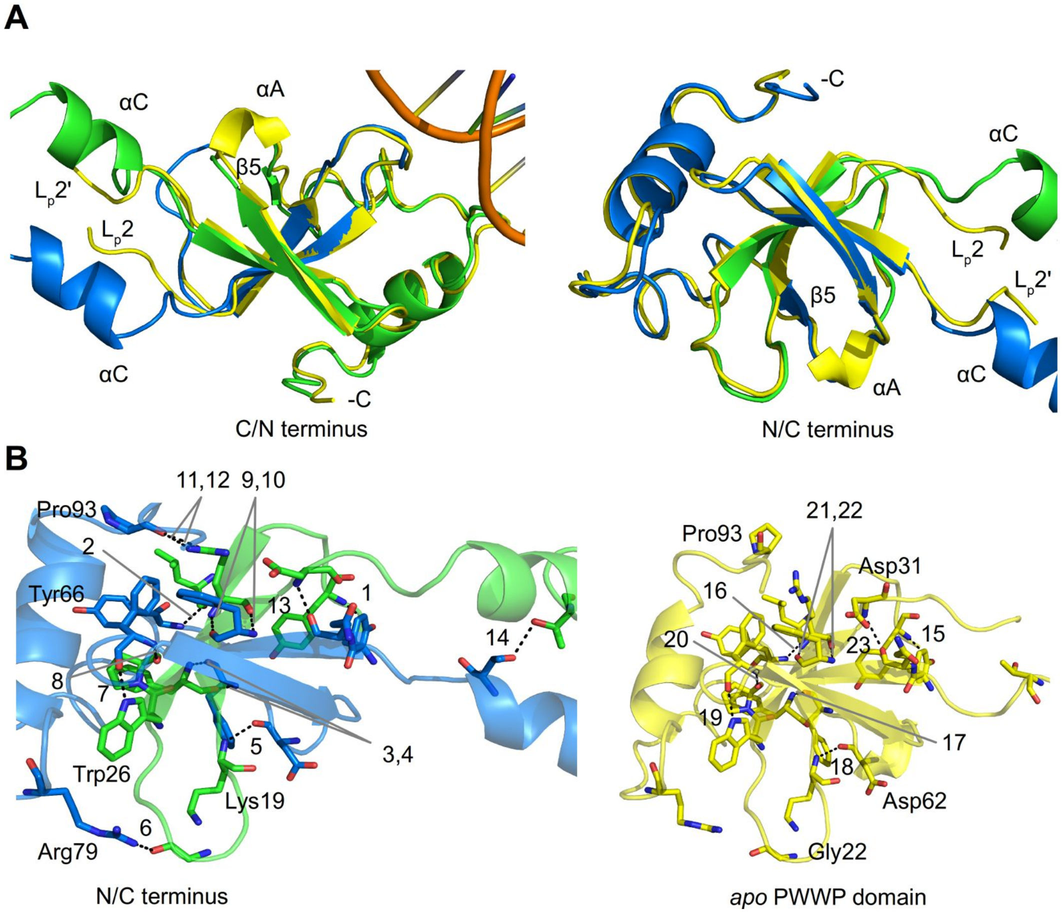

Figure S5. Structural comparisons between the *apo* PWWP domain and one swapped domain in the PWWP-*SMYD1* complex. (**A**) The superposition of the spatial arrangements of secondary structures of the *apo* PWWP domain (yellow) and the C/N and N/C termini in the PWWP-*SMYD1* complex (green: chain A; marine: chain B). The C/N or N/C terminus is similar to the *apo* PWWP domain except the loop2 region, αA, β5 and partial N-terminus and C-terminus. (**B**) Hydrogen-bonding residue pairs for domain swapping in the C/N terminus (green and marine) and those corresponding residues pairs in the *apo* PWWP domain (yellow). The pair residues are shown as sticks; the interactions are shown in the following numbers at Table S4. Each hydrogen bond is shown with dashed lines.

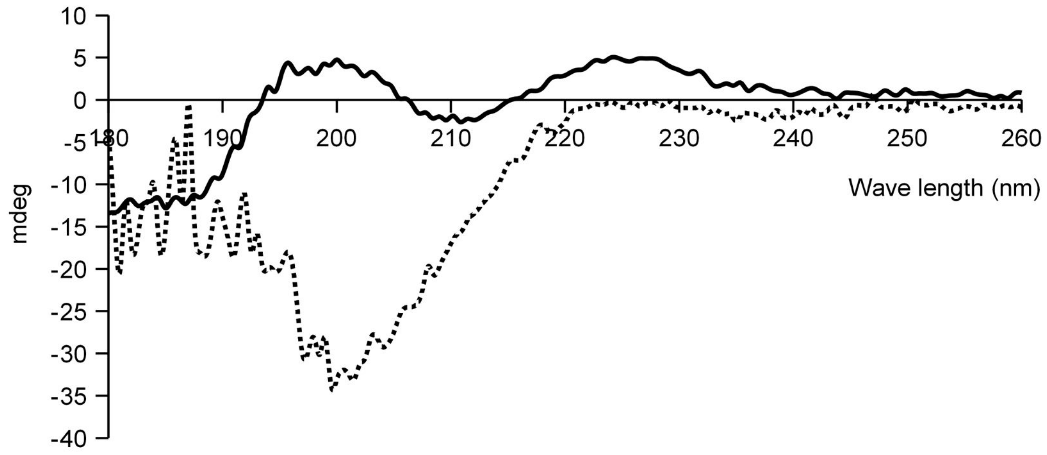

Figure S6. Determination of secondary structures of the full-length HDGF and the *apo* PWWP domain with SRCD. The spectrum of the *apo* PWWP domain (solid line) shows a β-strand dominant profile, whereas the HDGF (dashed line) reveals a profile of most random coils of the CD spectrum.

A

```
                        10        20        30        40        50
                         |         |    *   *  |         |         |
HDGF       1    --------MSRSNRQKEYKCGDLVFAKMKGYPHWPARIDEMPEA-----A    37
HDGF2      1    -------------MPHAFKPGDLVFAKMKGYPHWPARIDDIADG-----A    32
HDGF3      7    ---------------REYKAGDLVFAKMKGYPHWPARIDELPEG-----A    36
LEDGF      1    -------------MTRDFKPGDLIFAKMKGYPHWPARVDEVPDG-----A    32
MSH6      81    --------APAAPTSCDFSPGDLVWAKMEGYPWWPCLVYNHPFDGTFI-R   121
Pdp1      45    ------------SADDRLNFGDRILVKAPGYPWWPALLLRRKETKDSLNT    82
DNMT3B   206    EADSGDGDSSEYQDGKEFGIGDLVWGKIKGFSWWPAMVVSWKATS----K   251

                        60        70        80        90       100
                         |         |         |         |   # **  |
HDGF      38    VKSTANKYQVFFFGTHET-AFLGPKDLFPYEE-SKEKFGKP--NKRKGFS    83
HDGF2     33    VKPPPNKYPIFFFGTHET-AFLGPKDLFPYDK-CKDKYGKP--NKRKGFN    78
HDGF3     37    VKPPANKYPIFFFGTHET-AFLGPKDLFPYKE-YKDKFGKS--NKRKGFN    82
LEDGF     33    VKPPTNKLPIFFFGTHET-AFLGPKDIFPYSE-NKEKYGKP--NKRKGFN    78
MSH6     122    EKGKSVRVHVQFFDDSPTRGWVSKRLLKPYTG-SKSKEAQKGGHFYSAKP   170
Pdp1      83    NSSFNVLYKVLFFPDFNF-AWVKRNSVKPLLDSEIAKFLGSSKRKSKELI   131
DNMT3B   252    RQAMSGMRWVQWFGDGKF-SEVSADKLVALGL-FSQHFNLATFNKLVSYR   299

                       110       120       130       140       150
                         |         |         |         |         |
HDGF      84    EGLWEIENNPTVKASGY---------------------------------   100
HDGF2     79    EGLWEIQNNPHASYSAPPPVSS----------------------------   100
HDGF3     83    EGLWEIENNPGVKFTGYQAIQQQSSSETEGEG------------------   114
LEDGF     79    EGLWEIDNNPKVKFSSQQAATK----------------------------   100
MSH6     171    EILRAMQRADEALNKDKIKRLELAVCDEPS--------------------   200
Pdp1     132    EAYEASKTPPDLKEESSTDEEMDSLSAAE---------------------   160
DNMT3B   300    KAMYHALEKARVRAGKTFPSSPGDSLEDQLKPMLEWAHGGFKPTGIEGLK   349

HDGF            ------
HDGF2           ------
HDGF3           ------
LEDGF           ------
MSH6            ------
Pdp1            ------
DNMT3B   350    PNNTQP   355
```

B

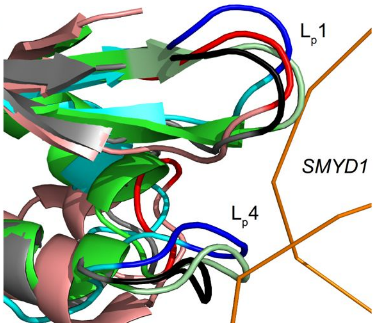

Figure S7. Sequence alignment and structure comparison of various PWWP domains. (**A**) The conserved PWWP motif is included in the black box. The DNA-binding residues in the HDGF PWWP-*SMYD1* complex are indicated with *, whereas the binding residues from the symmetry-related HDGF PWWP-*SMYD1* complex are marked with #. The conserved residues are highlighted with red (identical) and blue (low similarity) within the DNA-binding regions, loop1 ($_{19}$KMKG$_{22}$) and loop4 ($_{77}$NKRK$_{80}$). All those PWWP domains contain a highly conserved DNA-binding region, loop1 (especially residues Lys19 and Gly22). The other DNA-binding region,

loop4 shares a low similarity among those PWWP domains except the isoforms from the HDGF family (HDGF, HDGF2 and HDGF3). The involved DNA-binding residues detected by NMR chemical-shift perturbation are marked with yellow and cyan (mouse and human HDGF PWWP domain share the same sequence). The alignment was generated with Clustal W with manual adjustment[47]. (**B**) The superimposition of the DNA-binding regions among the crystal structure of the PWWP-*SMYD1* complex (gray), the NMR solution structure of the mouse HDGF PWWP domain (PDB entry: 2B8A, green), the NMR solution structure of the MSH6 PWWP domain (PDB entry: 2GFU, salmon) and the NMR solution structure of the LEDGF/p75 PWWP domain (PDB entry: 2M16, cyan). The DNA-binding regions in the PWWP-*SMYD1* complex are the loops (black) that are similar to other PWWP domains (palegreen, red and blue).

Reference

47. Thompson, J.D., Higgins, D.G. & Gibson, T.J. CLUSTAL W: improving the sensitivity of progressive multiple sequence alignment through sequence weighting, position-specific gap penalties and weight matrix choice. *Nucleic Acids Res.* **22**, 4673-4680 (1994).

No. pair interactions	Chain A	Chain B
1	Tyr10	Tyr45
2	Leu15	Tyr66
3, 4	Phe17	Phe64
5	Lys19	Asp62
6	Gly22	Arg79
7	Trp26	Phe50
8	Pro27	Phe50
9, 10	Arg29	Phe48
11, 12	Arg29	Pro93
13	Asp31	Gln46
14	Thr41	Thr41
15	Try45	Tyr10
16	Gln46	Asp31
17, 18	Phe48	Arg29
19	Phe50	Trp26
20	Phe50	Pro27
21	Asp62	Arg19
22, 23	Phe64	Phe17
24	Tyr66	Leu15
25	Arg79	Gly22
26, 27	Pro93	Arg29

Table S1. The numbers of pair interactions correspond to those in Fig. 2F.

Hydrogen-bonding residue pairs (The PWWP-*SMYD1* complex)		Distance (Å)	Hydrogen-bonding residues pairs (The *apo* PWWP domain)		Distance (Å)
Chain A	Chain B				
Tyr10	Tyr45 (OH)	3.0	Tyr10	Tyr45 (OH)	3.4
Leu15	Tyr66	3.0	Leu15	Tyr66	3.1
Phe17 (N) / (O)	Phe64 (O) / (N)	2.8 / 3.1	Phe17 (N)	Phe64 (O)	2.6
Lys19	Asp62	3.1	Lys19	Asp62	3.1
Gly22	Arg79	2.4	Trp26 (NE1)	Phe50 (O)	3.0
Trp26 (NE1)	Phe50 (O)	2.9	Pro27	Phe50 (N)	3.1
Pro27	Phe50 (N)	2.9	Arg29 (O) / (N)	Phe48 (N) / (O)	2.8 / 3.1
Arg29 (N) / (O)	Phe48 (O) / (N)	2.9 / 2.9	Asp31	Gln46 (O)	3.2
Arg29 (NE) / (NH1)	Pro93 (O)	3.2 / 3.4			
Asp31	Gln46	2.7			
Thr41	Thr41	3.1			
Tyr45 (OH)	Tyr10	3.0			
Gln46	Asp31	2.7			
Phe48 (O) / (N)	Arg29 (N) / (O)	2.9 / 2.9			
Phe50 (O)	Trp26 (NE1)	2.9			
Phe50 (N)	Pro27	2.9			
Asp62	Arg19	2.9			
Phe64 (O) / (N)	Phe17 (N) / (O)	2.7 / 3.2			
Tyr66	Leu15	3.2			
Arg79	Gly22	3.2			
Pro93 (O)	Arg29 (NH2) / (NE)	3.2 / 3.4			

Table S2. Hydrogen-bonding residue pairs of the swapped domain in the PWWP-*SMYD1* complex and the *apo* PWWP domain. The corresponding interaction distances are given.

Region	Chain A	Chain B	*apo*
	PWWP-*SMYD1* complex		PWWP domain
Hinge loop/αC	1.0	1.0	1.51
αA/β5	1.2 (1.2)[a]	1.11 (1.11)	1.13 (1.21)
DNA binding loops [b]	1.14 (1.14)	1.05 (1.06)	1.07/1.07 (1.16/1.15)
Domain-swapped residues	0.79 (0.79)	0.8 (0.79)	0.94 (1.01)

Table S3. Normalised *B*-factor values at the regions of structural differences between the PWWP-*SMYD1* complex and the *apo* PWWP domain. The normalised *B*-factor value for each region is calculated by dividing the average *B*-factor value for the region by the overall *B*-factor for the whole molecule.

[a]Values in parentheses for the normalised *B*-factor value for each region is calculated by dividing the average *B*-factor value for the region by the overall *B*-factor for the whole molecule except for the highly dynamic the hinge loop to avoid the influence.

[b]The normalized *B*-factors of DNA-binding loop4 (chain A) and loop1 (chain B) are presented, respectively, in the C/N terminus of the complex. The values of normalized *B*-factor from loops 4/1, respectively, are shown in the *apo* PWWP domain.

No. pair interactions	Chain A	Chain B
1, 15	Tyr10	Tyr45
2, 16	Leu15	Tyr66
3, 4, 17	Phe17	Phe64
5, 8	Lys19	Asp62
6	Gly22	Arg79
7, 19	Trp26	Phe50
8, 20	Pro27	Phe50
9, 10, 21, 22	Arg29	Phe48
11, 12	Arg29	Pro93
13, 23	Asp31	Gln46
14	Thr41	Thr41

Table S4. The numbers of pair interactions correspond to those in Fig. S5B.
